# Swab Testing to Optimize Pneumonia Treatment With Empiric Vancomycin: A Randomized Controlled Trial

**DOI:** 10.1093/cid/ciag295

**Published:** 2026-06-02

**Authors:** Jeffrey A Freiberg, Edward T Qian, Kylie G Nairon, Benjamin J Ereshefsky, Cassandra Hennessy, Grace Van Winkle, Ariel A Lewis, Joy M Justice, Justin K Siemann, Joanna L Stollings, Taylor M Rali, Frank E Harrell, Cheryl L Gatto, George E Nelson, Todd W Rice, for the Vanderbilt Center for Learning Healthcare, Gordon R Bernard, Robert S Dittus, Peter J Embi, Chad Fitzgerald, Robert E Freundlich, Cheryl L Gatto, Frank E Harrell, Paul A Harris, Tina Hartert, Catherine H Ivory, Ruth Kleinpell, Sunil Kripalani, Lee Ann Liska, Patrick Luther, Jay Morrison, Jill M Pulley, Edward T Qian, Kris Rehm, Todd W Rice, Russell L Rothman, Patti Runyan, Wesley H Self, Matthew W Semler, Robin Steaban, Cosby A Stone, Philip D Walker, Consuelo H Wilkins, Adam Wright, Autumn D Zuckerman

**Affiliations:** Division of Infectious Diseases, Department of Medicine, Vanderbilt University Medical Center, Nashville, Tennessee, USA; Vanderbilt Institute for Infection, Immunology and Inflammation, Vanderbilt University Medical Center, Nashville, Tennessee, USA; Department of Pathology, Microbiology, and Immunology, Vanderbilt University Medical Center, Nashville, Tennessee, USA; United States Department of Veterans Affairs Medical Center, Nashville, Tennessee, USA; Division of Allergy, Pulmonary, and Critical Care Medicine, Vanderbilt University Medical Center, Nashville, Tennessee, USA; Department of Anesthesiology, Vanderbilt University Medical Center, Nashville, Tennessee, USA; Department of Biomedical Informatics, Vanderbilt University Medical Center, Nashville, Tennessee, USA; Vanderbilt Institute for Clinical & Translational Research, Vanderbilt University Medical Center, Nashville, Tennessee, USA; Department of Pharmaceutical Services, Vanderbilt University Medical Center, Nashville, Tennessee, USA; Department of Biostatistics, Vanderbilt University Medical Center, Nashville, Tennessee, USA; Vanderbilt Institute for Clinical & Translational Research, Vanderbilt University Medical Center, Nashville, Tennessee, USA; Vanderbilt Institute for Clinical & Translational Research, Vanderbilt University Medical Center, Nashville, Tennessee, USA; Vanderbilt University School of Medicine, Nashville, Tennessee, USA; Vanderbilt Institute for Clinical & Translational Research, Vanderbilt University Medical Center, Nashville, Tennessee, USA; Department of Pharmaceutical Services, Vanderbilt University Medical Center, Nashville, Tennessee, USA; Medical Intensive Care Unit, Vanderbilt University Medical Center, Nashville, Tennessee, USA; Department of Biostatistics, Vanderbilt University Medical Center, Nashville, Tennessee, USA; Vanderbilt Institute for Clinical & Translational Research, Vanderbilt University Medical Center, Nashville, Tennessee, USA; Department of Emergency Medicine, Vanderbilt University Medical Center, Nashville, Tennessee, USA; Division of Infectious Diseases, Department of Medicine, Vanderbilt University Medical Center, Nashville, Tennessee, USA; Division of Allergy, Pulmonary, and Critical Care Medicine, Vanderbilt University Medical Center, Nashville, Tennessee, USA; Vanderbilt Institute for Clinical & Translational Research, Vanderbilt University Medical Center, Nashville, Tennessee, USA

**Keywords:** community-acquired pneumonia, antimicrobial stewardship, MRSA nasal colonization, ICU, *Staphylococcus aureus*

## Abstract

**Background:**

Fear of methicillin-resistant *Staphylococcus aureus* (MRSA) as a cause of community-acquired pneumonia (CAP) frequently leads to empiric vancomycin coverage. Data evaluating the use of MRSA polymerase chain reaction (PCR) nasal swab testing to guide vancomycin de-escalation is limited for patients in the intensive care unit (ICU).

**Methods:**

Swab Testing to Optimize Pneumonia Treatment With Empiric Vancomycin (STOP-Vanc) is a pragmatic, prospective, single-center, non-blinded randomized trial in which adult ICU patients with suspicion of CAP were randomized 1:1 to receive usual care either with (intervention) or without (control) the addition of MRSA nares PCR testing following ICU admission. The primary outcome was vancomycin-free hours alive, defined as the expected number of hours alive and free of vancomycin use within the first 7 days of trial enrollment as estimated using a longitudinal proportional odds state transition model adjusted for baseline covariates.

**Results:**

A total of 277 adult ICU patients were randomized. Methicillin-resistant *Staphylococcus aureus* PCR nasal swab testing had a negative predictive value (NPV) of 98.9% in the intervention arm. The primary endpoint, vancomycin-free hours alive, was 105.7 in the control arm and 109.7 in the intervention arm (adjusted difference, 4 hours; 95% CI, −9.5–18.2; *P* = .458).

**Conclusions:**

Despite MRSA PCR nasal swab testing demonstrating a high NPV in this critically ill population, MRSA PCR nasal swab testing did not decrease the duration of vancomycin use or 30-day mortality among ICU patients with suspected CAP. Additional clinician education and antimicrobial stewardship interventions might be needed to reduce vancomycin use in this patient population.

**Clinical Trials Registration:**

ClinicalTrials.gov NCT06272994 (STOP-Vanc).

## Teaser

In this pragmatic randomized trial of 277 adults, vancomycin-free hours alive in the first 7 days of trial enrollment did not significantly differ between patients who received MRSA PCR nasal swab testing plus usual care versus usual care alone.

Severe community-acquired pneumonia (CAP) is a leading cause of mechanical ventilation and is associated with a high mortality rate [1]. Empiric antibiotic regimens frequently include intravenous vancomycin for methicillin-resistant *Staphylococcus aureus* (MRSA) coverage in critically ill patients with suspected CAP, regardless of the patient's risk factors for an MRSA infection [2,3]. Despite this, the incidence of CAP due to MRSA is low, accounting for less than 5% of all infections, even in an ICU setting [4, 5]. The 2019 American Thoracic Society/Infectious Diseases Society of America CAP guidelines only recommend the addition of empiric anti-MRSA coverage if patients have a history of MRSA colonization or MRSA infection in the past year, or if they were hospitalized and received parenteral antibiotics in the last 90 days [6]. Empiric vancomycin use in patients with CAP is associated with increased mortality, acute kidney injury (AKI), and a higher rate of secondary infections [7–9]. Furthermore, the use of vancomycin can select for antibiotic-resistant organisms, leading to further treatment challenges [10]. Thus, antimicrobial stewardship strategies to reduce unnecessary empiric vancomycin are an attractive avenue to improve patient safety and health outcomes.

Lack of MRSA colonization of the anterior nares has a high negative predictive value (NPV) for MRSA pneumonia with the absence of MRSA on nasal swabs corresponding to a NPV of >95% in pneumonia [11–13]. While blood and sputum culture methods are the conventional standard in infectious diseases, they are labor intensive and necessitate long turn-around times for final results [11]. Testing that utilizes polymerase chain reaction (PCR) amplification to detect MRSA DNA allows for rapid results, increased sensitivity to low bacterial load, and potentially earlier de-escalation of antimicrobial therapy [2, 14]. Multiple retrospective studies and a recent randomized controlled trial (RCT) have found reduced durations of anti-MRSA therapy after the implementation of MRSA PCR nasal swab testing [15–25]. However, prospective RCTs of MRSA PCR nasal swab testing in critically ill patients admitted with CAP are still lacking.

In this pragmatic, randomized clinical trial, we evaluated the effectiveness of MRSA PCR nasal swab testing added to usual care in reducing unnecessary vancomycin use, 30-day all-cause mortality, and other clinical and antimicrobial stewardship outcomes in intensive care unit (ICU) patients with suspected CAP receiving vancomycin.

## METHODS

### Trial Design and Oversight

Between April 2024 and January 2025, this pragmatic, prospective, randomized trial was conducted in a tertiary care, academic hospital ICU setting. A diverse patient population was studied to assess the comparative effectiveness of usual care plus PCR-based MRSA nasal swab testing versus usual care alone on vancomycin-free hours alive and 30-day all-cause mortality among adult patients with suspected CAP prescribed empiric vancomycin. This investigator-initiated trial was approved by the institutional review board (IRB) under a waiver of informed consent as a minimal risk study. Institutional review board approval (IRB #231724) and ClinicalTrials.gov (NCT06272994) registration were obtained prior to participant enrollment, and the trial protocol and statistical analysis plan were made public before enrollment ended [26]. The trial protocol and statistical analysis plan can be found in Supplement 1.

### Patient Population and Randomization

Adult (≥18 years old) patients admitted to the medical ICU (MICU) were included if, within 24 hours of their admission to the MICU, they had all three of the following: (1) either an order for vancomycin or pharmacy monitoring of vancomycin, (2) an order for a respiratory culture or respiratory infection selected as the indication for vancomycin, and (3) no prior receipt of topical nasal decolonization. Hospital policy required that universal mupirocin decolonization be ordered routinely for all patients upon admission to the MICU. However, patients could still be enrolled if mupirocin was ordered but not yet administered. All patients admitted to the MICU were automatically screened for inclusion whenever their electronic health record (EHR) was opened. Patients with a hospital stay of more than 48 hours prior to MICU transfer were excluded, as well as those known to be incarcerated. A silent clinical decision support tool within the EHR screened patients for eligibility and, if met, electronically enrolled and randomized the patient for the study. Patients were randomized in a 1:1 ratio using simple randomization within the EHR.

### Study Arms

Upon enrollment, the study team was notified of participants randomized to the intervention arm, and an MRSA PCR nasal swab testing order was placed by the study team. If the test was negative, the participant's provider was notified via an automated pager alert, sent to the team pager for the patient's current primary inpatient team. The pager alert included the negative results and a targeted antimicrobial stewardship message prompting the discontinuation of vancomycin. If the test returned positive or was inconclusive, no pager alert was sent. Methicillin-resistant *Staphylococcus aureus* PCR nasal swab test results were also available within the EHR. In both arms, the decision to start or stop vancomycin was ultimately left to the discretion of the treating team, as were all other interventions. In the usual care group, no order was placed by the study team for MRSA PCR nasal swab testing. Critical care pharmacists who routinely provide antimicrobial stewardship guidance in the ICU were part of patients’ regular care in both groups, but they provided additional recommendations and guidance that incorporated, when available, MRSA PCR nasal swab testing results. Given the pragmatic nature of this trial, providers caring for participants in the usual care arm were not restricted from ordering MRSA PCR nasal swab testing; however, testing is only done with a specific order from a provider at this hospital and not automatically performed on admission. Pager alerts were not sent for participants in the control arm even if they had testing performed.

### Data Collection

All data in this study were generated during routine patient care and extracted electronically from the EHR or by manual chart review. Data included demographic and baseline comorbidity information; laboratory values; antibiotic ordering and administration records; MRSA PCR test timing and results; blood and sputum culture results; dates of admission, transfer, and discharge; and discharge status.

### Outcomes

The primary outcome for this study was the estimated expected number of vancomycin-free hours alive in the 7 days (168 hours) following study enrollment based on a 3-state longitudinal proportional odds state transition model [26]. This approach was chosen to account for the anticipated high mortality rate in the study population. Secondary outcomes included time alive off vancomycin in the 7 days (168 hours) following study enrollment and 30-day all-cause mortality. For the secondary outcome of time alive off vancomycin, this was the median number of actual hours and was included to provide a second more traditional method of accounting for mortality when calculating vancomycin duration. Vancomycin durations were calculated from the start time of the administration of an initial dose of vancomycin to the start time of the last dose received. If a subsequent order for vancomycin was placed after a 24-hour period without an active order for vancomycin or a pharmacy consult for vancomycin, it was considered a re-start of vancomycin, and a separate duration interval was calculated. Prespecified exploratory, implementation, and fidelity endpoints were collected as previously published [26].

### Statistical Analysis

Details regarding the initial sample size calculation for this trial have been published previously [26]. Assuming a 2-sided alpha of 0.05 and an estimated mortality rate of 22% at 8 days, a sample size of 106 patients per arm would detect a 24-hour increase in vancomycin-free hours alive with 90% power. Near the midpoint of the trial, we determined that approximately 12% of patients had MRSA PCR nasal swab testing ordered prior to enrollment, and a further 3% of patients had been co-enrolled in another competing study that was also investigating a molecular test to reduce empiric vancomycin use. Based on these rates, we enrolled additional patients as appropriate until the lowest enrolled arm reached 106 patients without counting patients in these 2 populations.

The primary analysis cohort included all patients who were enrolled in the study, analyzed according to the arm to which they were randomized (intention-to-treat). A secondary analysis was performed with the adjusted cohort (modified study population) excluding patients who had MRSA nasal swab testing prior to enrollment, patients enrolled in the competing study, and one patient who was enrolled in the usual care arm despite never meeting inclusion criteria. The primary outcome was assessed using a first-order Markov ordinal longitudinal proportional odds state transition model adjusted for covariates and calculated on 3 states: alive and off vancomycin, alive and on vancomycin, or death [26]. In this model, the treatment effect is an odds ratio (OR) for transitioning to a “worse” state, with alive and not on vancomycin being the best state and deceased being the worst state [27]. An OR > 1 indicates a higher odds of vancomycin use or death in the control group. From the OR, in conjunction with the modeled effect of time, the probability of being in a given state at a given hour based on the past hour's state can be estimated. From this, the probability of being in each of the 3 states can be calculated as a function of time, treatment, and baseline covariates, thus providing an estimated mean vancomycin-free hours alive. Secondary and exploratory outcomes were assessed with generalized linear models and adjusted for covariates, except for antibiotic durations, which were assessed using the same approach as the primary outcome. In these secondary and exploratory outcomes, statistical significance was calculated using a Wilcoxon–Mann–Whitney nonparametric test, a chi-squared test, or a Cox proportional hazards model using a competing risk of mortality.

## RESULTS

### Study Participants

**Figure 1.**
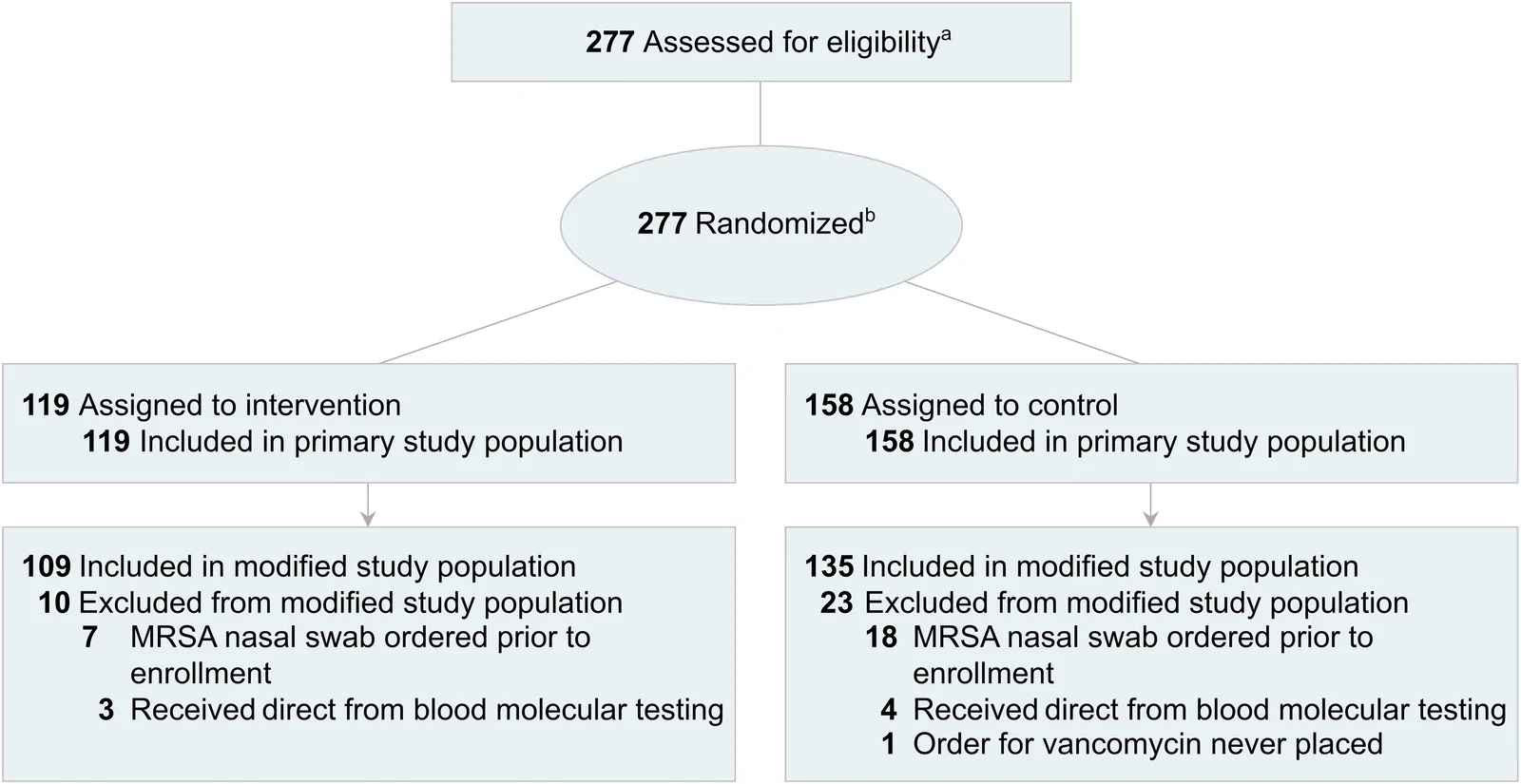
Flow of participants through the Swab Testing to Optimize Pneumonia Treatment With Empiric Vancomycin (STOP-Vanc) trial. ^a^All patients admitted to the ICU within 48 h of hospitalization were continuously screened by an algorithm in the electronic health record for the presence of inclusion criteria (age ≥ 18 y, admission to the intensive care unit [ICU] from the emergency department or a hospital ward, no receipt of topical nasal decolonization prior to enrollment, active order for vancomycin or a pharmacokinetics consult for vancomycin dosing, and either a respiratory indication for vancomycin or an order for a respiratory culture). ^b^Randomization was carried out using the randomization mechanism embedded within the electronic health record, which occurs completely independent of any participant variables. Randomization ratio was set 1:1 between arms without stratification.

There were 277 participants included in the primary study population (Figure 1). A total of 158 participants were randomized to the control arm, and 119 were randomized to the intervention arm. The modified study population (which excluded participants with a MRSA PCR nasal swab test ordered prior to enrollment, participants with a direct blood molecular test result, and one participant who never had an order for vancomycin placed) contained 244 participants with 135 in the control arm and 109 in the intervention arm. The reasons for exclusion of participants are included in Figure 1. Baseline demographics and admission characteristics were similar between study arms for both the full study population and the modified study population (Table 1). The median Elixhauser Comorbidity Index was 13 (Interquartile Range [IQR] 2–23) and the median Sequential Organ Failure Assessment (SOFA) score on admission to the ICU was 7 (IQR 4–10). Of the 277 participants, 235 (85%) required supplemental oxygen on ICU admission with 79 (29%) requiring mechanical ventilation. Fifty-three participants (19%) had chronic kidney disease with 10 (4%) requiring hemodialysis at baseline. Immunocompromising conditions were present in 35 (13%) participants. At least 1 blood or respiratory culture was collected for 271 (98%) participants. Despite all participants having had an order for a respiratory culture, only 173 (62%) had a respiratory culture successfully collected. Overall, 15 participants had MRSA isolated from blood or respiratory cultures either prior to enrollment or in the 7 days following enrollment, with a higher percentage having MRSA infections (8 participants, 7%) in the intervention group than the control group (7 participants, 4%).

**Table 1.** Baseline Demographics and Admission Characteristics

	No. (%)					
	Full Study Population			Modified Study Population^a^		
Characteristic	All (n = 277)	Intervention (n = 119)	Control (n = 158)	All (n = 244)	Intervention (n = 109)	Control (n = 135)
Participant demographics						
Age, median (IQR), y	62 (51–71)	62 (51–69)	62 (51–72)	62 (51–70)	62 (51–70)	61 (51–71)
Sex						
Female	100 (36)	42 (35)	58 (37)	94 (39)	40 (37)	54 (40)
Male	177 (64)	77 (65)	100 (63)	150 (61)	69 (63)	81 (60)
Race^b^						
White	219 (79)	96 (81)	123 (78)	192 (79)	89 (82)	103 (76)
Black/African American	42 (15)	16 (13)	26 (16)	38 (16)	15 (14)	23 (17)
Other	5 (2)	3 (3)	2 (1)	5 (2)	3 (3)	2 (1)
Unknown	18 (6)	7 (6)	11 (7)	16 (7)	6 (6)	10 (7)
Ethnicity						
Hispanic	13 (5)	7 (6)	6 (4)	12 (5)	6 (6)	6 (4)
Non-Hispanic	244 (88)	106 (89)	138 (87)	213 (87)	98 (90)	115 (85)
Unknown	20 (7)	6 (5)	14 (9)	19 (8)	5 (5)	14 (10)
Elixhauser Comorbidity Index, median (IQR)	13 (2–23)	15 (4–24)	12 (2–21)	13 (2–23)	16 (5–26)	11 (0–21)
Baseline CKD present	53 (19)	28 (24)	25 (16)	47 (19)	26 (24)	21 (16)
Baseline ESRD	10 (4)	3 (3)	7 (4)	10 (4)	3 (3)	7 (5)
immunocompromised	35 (13)	17 (14)	18 (11)	33 (14)	17 (16)	16 (12)
HIV	11 (4)	5 (4)	6 (4)	11 (5)	5 (5)	6 (4)
Solid organ transplant	21 (8)	11 (9)	10 (6)	19 (8)	11 (10)	8 (6)
Stem cell transplant	4 (1)	2 (2)	2 (1)	4 (2)	2 (2)	2 (1)
Body mass index, mean (SD) kg/m^2c^	27 (9)	28 (8)	27 (9)	28 (9)	28 (8)	28 (9)
Admission characteristics						
SOFA score on admission to ICU, median (IQR)	7 (4–10)	6 (4–10)	7 (5–11)	7 (4–10)	6 (4–9)	7 (5–11)
Requiring supplemental oxygen	235 (85)	101 (85)	134 (85)	207 (85)	92 (84)	115 (85)
Requiring mechanical ventilation	79 (29)	34 (29)	45 (29)	74 (30)	31 (28)	43 (32)
Requiring vasopressor support	125 (45)	46 (39)	79 (50)	114 (47)	43 (39)	71 (53)
Reference creatinine, median (IQR) mg/dL^d^	1.2 (0.8–2.1)	1.2 (0.8–2.1)	1.2 (0.8–2.0)	1.2 (0.8–2.1)	1.2 (0.8–2.1)	1.2 (0.8–2.0)
Blood or respiratory cultures collected	271 (98)	116 (97)	155 (98)	238 (98)	106 (97)	132 (98)
Number of blood or respiratory cultures collected, median (IQR)	3 (2–4)	3 (2–4)	3 (2–4)	3 (2–4)	3 (2–4)	3 (2–4)
Blood cultures collected	261 (94)	113 (95)	148 (94)	230 (94)	104 (95)	126 (93)
Number of blood cultures collected, median (IQR)	2 (2–4)	2 (2–4)	2 (2–3)	2 (2–4)	2 (2–4)	2 (2–4)
Respiratory cultures collected	173 (62)	72 (61)	101 (64)	153 (63	64 (59)	89 (66)
Number of respiratory cultures collected, median (IQR)	1 (0–1)	1 (0–1)	1 (0–1)	1 (0–1)	1 (0–1)	1 (0–1)
MRSA PCR nasal swab result in the previous 30 d^e^	21 (8)	10 (8)	11 (7)	16 (7)	9 (8)	7 (4)
Blood or respiratory cultures with MRSA^f^	15 (5)	8 (7)	7 (4)	15 (6)	8 (7)	7 (5)

^a^An adjusted cohort that excludes patients who had MRSA PCR nasal swab testing prior to enrollment, were enrolled in a competing study, or were enrolled despite never meeting inclusion criteria.

^b^Percentages do not necessarily equal 100% as race categories were not mutually exclusive.

^c^Unable to calculate BMI for 2 participants in the control group due to missing data.

^d^Reference creatinine was defined as the most recent creatinine obtained since admission but prior to the administration of the first dose of vancomycin. If no such value existed, then the most immediate creatinine following the administration of vancomycin was used.

^e^Includes MRSA PCR nasal swabs if they were ordered in the 30 d prior to enrollment and if they occurred in a separate hospital admission from the study admission.

^f^Among cultures collected either prior to enrollment or within the first 7 d following enrollment.

### Intervention Fidelity

All 119 participants in the intervention arm had MRSA PCR nasal swab testing ordered and collected, while 31 participants (20%) in the control arm had MRSA PCR nasal swab testing ordered by a clinical team either prior to enrollment or in the 7 days following enrollment (Table 2). The timing of MRSA PCR nasal swab ordering in both the control and intervention arms is shown in Supplementary Figure 1 in Supplement 2. Methicillin-resistant *Staphylococcus aureus* PCR nasal swab test results were available in 30 participants in the control arm (19%) and 118 participants in the intervention arm (99%). The median time from study enrollment to MRSA PCR nasal swab test results being available in the EHR was 6 hours (IQR 5–8). Methicillin-resistant *Staphylococcus aureus* PCR nasal swab testing was positive in 22 (18%) participants in the intervention arm, and providers caring for 89 out of the 96 participants (93%) with negative tests received the intended pager alert containing antimicrobial stewardship guidance. The proportion of patients receiving each aspect of the intervention is shown in Supplementary Figure 2 in Supplement 2.

**Table 2.** Fidelity and Performance of MRSA Nasal Swab PCR Testing

	No. (%)	
Measure	Intervention (n = 119)	Control (n = 158)
Fidelity endpoints		
Subjects with MRSA nasal swab ordered^a^	119 (100)	31 (20)
MRSA nasal swab ordered prior to enrollment	7 (6)	18 (58)
MRSA nasal swab collected after start of mupirocin administration	19 (16)	10 (33)
Subjects with MRSA nasal swab result	118 (99)	30 (19)
No MRSA detected	96 (81)	28 (93)
MRSA detected	22 (19)	2 (7)
Time from enrollment to ordering of swab, median (IQR), min	21 (10–46)^b^	N/A
Time from ordering of swab to collection of swab, median (IQR), min	55 (24–111)	43 (23–107)
Time from collection of swab to results being available in EHR, median (IQR), min^c^	246 (198–306)	250 (225–343)
Time from enrollment to results being available in EHR, median (IQR), h	6 (5–8)	N/A
Pager alert indicated	96 (81)	N/A
Pager alert sent	89 (93)	N/A
Pager alert indicated but not sent	7 (7)	N/A
Test results		
True positives	7 (6)	…
False positives	14 (12)	…
False negatives	1 (1)	…
True negatives	93 (78)	…
No corresponding blood or respiratory culture	3 (3)	…
No PCR testing result	1 (1)	…
Test performance (n = 115)^d^		
Sensitivity (95% CI)	0.875 (0.529–0.994)	…
Specificity (95% CI)	0.869 (0.792–0.920)	…
PPV (95% CI)	0.333 (0.172–0.546)	…
NPV (95% CI)	0.989 (0.942–1.000)	…

^a^Only includes MRSA PCR nasal swabs ordered from the time of admission to 7 d after enrollment.

^b^Time from enrollment to ordering of swab only calculated for subjects with swabs ordered after enrollment, n = 112.

^c^Time from collection of swab to results being available in the EHR is only calculated for subjects with MRSA nasal swab results, n = 118 for the intervention group and n = 30 for the control group.

^d^The presence of MRSA in a blood culture or respiratory culture collected either prior to enrollment or within the first 7 d following enrollment was used as a reference for determining test performance. Subjects were excluded from the analysis if no cultures with results existed.

### Primary Outcome

**Figure 2.**
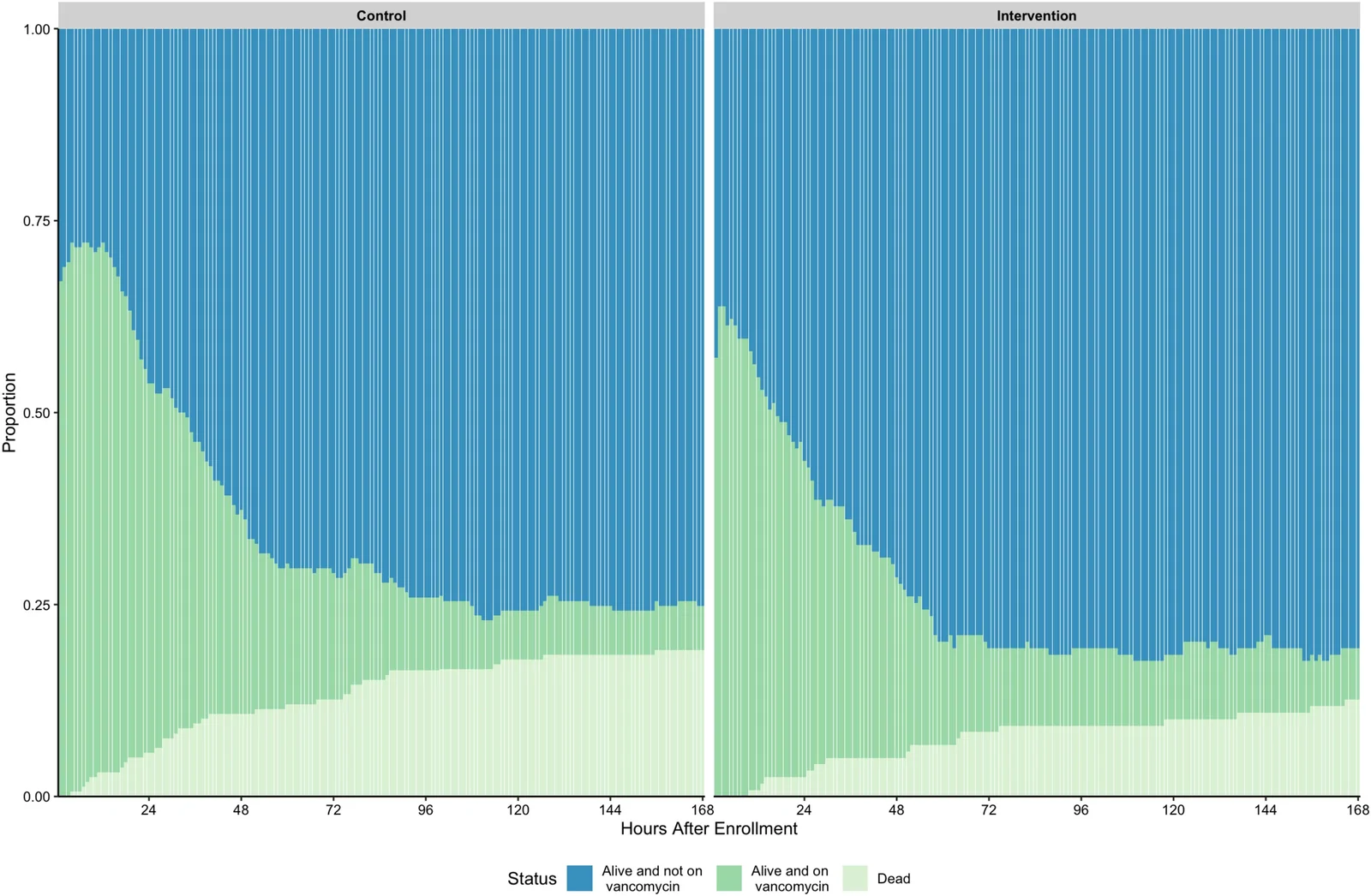
Proportion of subjects occupying a given state over time based on treatment arm in full study population. The relative proportions of the total number of subjects are displayed over cumulative time postenrollment as patients occupying 1 of 3 states: (1) alive and not on vancomycin (blue), (2) alive and on vancomycin (dark green), or (3) deceased (light green).

The crude proportion of participants occupying a given state over the first 168 hours (7 days) following enrollment are shown for each arm in Figure 2 (state occupancy data for the modified study population is shown in Supplementary Figure 3 in Supplement 2). Among the full study population, when adjusted for the prespecified baseline covariates of age, baseline creatinine, immunocompromised status, and SOFA score on ICU admission, the intervention arm had a mean of 109.7 vancomycin-free hours alive versus 105.7 in the control arm for a difference of 4 hours (95% CI, −9.5–18.2; *P* = .458) (Table 3). The modified study population similarly had a mean of 106.3 vancomycin-free hours alive in the intervention arm versus 103.0 in the control arm. There were no differential treatment effects based on any of the prespecified baseline covariates (Supplementary Table 1 in Supplement 2).

**Table 3.** Study Outcomes

A. Full Study Population					
	No. (%)				
Outcome	Intervention (n = 119)	Control (n = 158)	Difference (95% CI)	Odds Ratio (95% CI)	*P* value
Primary outcome					
Treatment transition odds ratio^a^	…	…	…	1.070 (0.895–1.278)	.459
Estimated expected number of vancomycin-free hours alive, h^b,c^	109.7	105.7	4.0 (−9.5–18.2)	…	.458
Estimated expected number of hours receiving vancomycin, h^b,c^	38.6	40.4	−1.8 (−8.4–4.9)	…	
Estimated expected number of hours alive, h^b,c^	148.3	146.1	2.3 (−6.0–10.1)	…	
Secondary outcomes					
Time alive off vancomycin, median (IQR), h^b,d^	138 (107–165)	128 (71–154)	12.6 (−0.01–25.2)	…	.049
30-d all-cause mortality	31 (26)	56 (35)	…	0.701 (0.408–1.204)	.124
Exploratory outcomes					
AKI^e^	34 (29)	49 (32)	…	0.926 (0.538–1.595)	.677
In-hospital all-cause mortality	23 (19)	42 (27)	…	0.716 (0.395–1.300)	.205
Mean length of stay, d	12.1	10.1	…	0.853 (0.650–1.119)	.250
Mean ICU length of stay, d	4.9	4.9	…	1.055 (0.812–1.372)	.687
Mean IV antibiotic-free days alive, d^f^	6.46	5.99	0.47 (−0.51–1.56)	1.118 (0.812–1.502)	.329
Mean time on IV antibiotics, d^f^	5.35	5.55	−0.19 (−0.55–0.00)	1.118 (0.827–1.473)	
Mean antibiotic-free days alive, d^f^	4.65	4.32	0.33 (−0.05–2.31)	1.085 (0.961–1.845)	.483
Mean time on antibiotics, d^f^	7.05	7.18	−0.13 (−0.76 to −0.14)	1.085 (1.045–1.837)	
Mean ventilator-free days, d^f^	10.99	10.15	…	1.428 (0.896–2.277)	.135
Receipt of alternative anti-MRSA antibiotics^g^	4 (3)	4 (3)	…	1.167 (0.271–5.031)	.964

B. Modified Study Population					
	No. (%)				
Outcome	Intervention (n = 109)	Control (n = 135)	Difference (95% CI)	Odds Ratio (95% CI)	*P* value
Primary outcome					
Treatment transition odds ratio^a^	…	…	…	1.057 (0.876–1.275)	.563
Estimated expected number of vancomycin-free hours alive, h^b,c^	106.3	103.0	3.3 (−14.4–19.8)	…	.563
Estimated expected number of hours receiving vancomycin, h^b,c^	40.6	42	−1.5 (−8.6–4.9)	…	
Estimated expected number of hours alive, h^b,c^	146.9	145.0	1.9 (−6.2–9.9)	…	
Secondary outcomes					
Time alive off vancomycin, median (IQR), h^b,d^	137 (104–162)	127 (67–153)	11.4 (−2.2–25.1)	…	.100
30-d all-cause mortality	29 (27)	49 (36)	…	0.694 (0.391–1.232)	.140
Exploratory outcomes					
AKI^e^	31 (29)	38 (30)	…	1.092 (0.606–1.969)	1.000
In-hospital all-cause mortality	22 (20)	38 (28)	…	0.720 (0.386–1.341)	.198
Mean length of stay, d	12.3	10.2	…	0.870 (0.650–1.163)	.347
Mean ICU length of stay, d	4.9	5.0	…	1.093 (0.826–1.448)	.534
Mean IV antibiotic-free days alive, d^f^	6.27	5.96	0.31 (−0.94–1.67)	1.076 (0.798–1.493)	.542
Mean time on IV antibiotics, d^f^	5.41	5.54	−0.12 (−0.62–0.38)	1.076 (0.795–1.445)	
Mean antibiotic-free days alive, d^f^	4.52	4.34	0.17 (−0.95–1.25)	1.044 (0.788–1.375)	.726
Mean time on antibiotics, d^f^	7.06	7.13	−0.07 (−0.51–0.39)	1.044 (0.783–1.387)	
Mean ventilator-free days, d^f^	10.89	9.93	…	1.485 (0.908–2.429)	.115
Receipt of alternative anti-MRSA antibiotics^g^	4 (3)	4 (4)	…	1.107 (0.257–4.782)	1.000

^a^Odds ratio of >1 indicates higher odds of transitioning to a “worse” state in the control group in the 3-state model with alive and not on vancomycin being the best state and deceased being the worst state.

^b^Value out of the first 168 h (7 d) following enrollment.

^c^Estimated expected number of hours based on a patient's odds of transitioning from their current state to a worse state using a 3-state proportional-odds state transition model. Estimated expected values denote model-based estimates of the mean [26].

^d^Median number of actual hours alive and free of vancomycin.

^e^Defined as an increase in creatinine to ≥1.5 times the reference creatinine level or an increase by ≥0.3 mg/dL within 14 d of study enrollment. Excludes participants who had end-stage renal disease (ESRD) prior to study enrollment.

^f^Value out of the first 14 d following enrollment.

^g^Includes any antibiotics considered to be appropriate options for the treatment of MRSA pneumonia (as prespecified in the study protocol), regardless of the actual reason for their use during the study.

### Secondary and Exploratory Outcomes

The median time alive off vancomycin was 138 hours in the intervention arm and 128 hours in the control arm. After adjusting for baseline covariates, the estimated difference in the time alive off vancomycin was 12.6 hours (95% CI, −.01–25.2) (Table 3). The distribution of time alive off vancomycin for study participants is shown in Supplementary Figure 4 in Supplement 2. The 30-day all-cause mortality was 26% in the intervention group compared to 35% in the control group (OR, 0.70; 95% CI, .41–1.20) (Table 3). Kaplan–Meier survival curves for both the full study population and the modified study population are shown in Supplementary Figure 5 in Supplement 2.

Acute kidney injury, length of stay, ICU length of stay, IV and total antibiotic-free days alive, median ventilator-free days, and receipt of nonvancomycin anti-MRSA antibiotics were all similar between the two groups (Table 3). For ICU and total hospital length of stay, competing risk analysis data is shown in Supplementary Figure 6 in Supplement 2. Crude state occupancy data for the outcomes of antibiotic-free days alive and IV antibiotic-free days alive are shown in Supplementary Figure 7 in Supplement 2. Out of the 118 participants with MRSA PCR nasal swab test results available in the intervention arm, 115 also had corresponding blood or respiratory cultures available for comparison, with 1 false negative and 93 true negatives for an overall NPV of 98.9% (95% CI, 94.2%–100.0%) (Table 2). Concordance between MRSA PCR nasal swab test results and blood or respiratory culture results is shown in Supplementary Table 2 in Supplement 2. For the 148 patients (118 intervention arm, 30 control arm) with MRSA PCR nasal swab test results, the time from result to discontinuation of the initial vancomycin order is shown in Supplementary Figure 8 in Supplement 2.

## DISCUSSION

Despite the inclusion of a recommendation for MRSA PCR nasal swab testing as an antimicrobial stewardship intervention in CAP guidelines, it had not been tested in a prospective, randomized trial in a critically ill patient population. This study allowed for the successful early randomization of MICU patients by utilizing a unique design where screening, enrollment, and randomization occurred entirely within the EHR. Patients in the intervention arm received MRSA PCR nasal swab testing early during the period of empiric vancomycin therapy with rapid reporting of test results and automated antimicrobial stewardship guidance. Among the other strengths of this study was the fact that all subjects in the intervention arm had an MRSA PCR nasal swab test collected and all but 1 patient received a result.

Despite this, the study found little evidence for a reduction in the duration of empiric vancomycin use. This result was unexpected and contrary to the multitude of observational studies and a prior RCT, which showed large reductions in anti-MRSA antibiotic use through the implementation of MRSA PCR nasal swab testing [15–25]. The mean number of hours receiving vancomycin for the control group in this study, 40.4 hours, was approximately half the duration of vancomycin used in the control group of prior observational studies [15–24]. In the only other RCT studying the use of MRSA PCR nasal swabs to de-escalate antibiotics, the control group had a mean anti-MRSA antibiotic duration of greater than 180 hours, despite that study having a similar MRSA infection rate and lower MRSA colonization rate [19]. The short duration of vancomycin utilization even in the absence of an intervention in our study was likely a combination of the study's pragmatic design, which relied on early enrollment of patients based on initial suspicion of CAP, and the existing practice of ICU pharmacist-supported antimicrobial stewardship within the MICU in which the study was performed [2]. Prior to and throughout this trial, pharmacists embedded within the ICU provided daily face-to-face evidence-based guidance regarding antibiotic de-escalation based on culture results or the lack of growth from cultures [2]. This established practice of direct pharmacist involvement and early de-escalation reduced the window in which to effect change as patients determined to have alternative diagnoses besides CAP would have been targeted for rapid vancomycin de-escalation, even in the control group. This is supported by a post hoc analysis of the subgroup of participants who were ultimately determined to have bacterial pneumonia based on their encounter billing diagnoses (Supplementary Tables 3 and 4 in Supplement 2). In this subgroup, the difference between the median time alive off vancomycin in the control and intervention arms was 18.9 hours, compared with 12.6 hours in the full study population (Supplementary Table 5 in Supplement 2). Interestingly, in this post hoc pneumonia subgroup, the intervention arm had lower in-hospital (10/58, 17%) and 30-day mortality (13/58, 22%) versus the control group (26/75, 35% and 31/75, 41%, respectively) (Supplementary Table 5 in Supplement 2). As this trial was designed to study patients with suspicion for CAP, and an analysis of patients with definitive diagnoses of pneumonia was not preplanned, these findings should only be treated as hypothesis generating. However, the mortality difference between the control and intervention arms in this subgroup warrants further study.

At an estimated cost of around $50 per MRSA PCR swab, broad use of this test is unlikely to be cost-effective, given the lack of a clear improvement in antibiotic utilization. However, this trial supports the safety and fidelity of the use of MRSA PCR nasal swab testing in an ICU population. There was a single false-negative test in the intervention group for a NPV of 98.9%, in line with other studies regarding the use of this test in CAP [12]. The false-negative test occurred in a patient who was identified to have MRSA bacteremia secondary to pelvic osteomyelitis prior to the MRSA PCR nasal swab testing result being available, so vancomycin was never discontinued. There were no false negatives among the 19 participants (16%) who received mupirocin prior to the collection of MRSA PCR nasal swab tests, consistent with a recently published retrospective study demonstrating that the use of MRSA PCR testing in the 7 days after the start of mupirocin decolonization is noninferior to swab testing prior to decolonization [28]. Given that there may be potential benefits to the subgroup of patients ultimately diagnosed with bacterial pneumonia (Supplementary Table 5 in Supplement 2) and no signal for harm identified in the broader population with suspicion for pneumonia, the potential for clinical benefit may justify the use of MRSA PCR nasal swab testing, particularly in settings without strong existing antimicrobial stewardship efforts.

### Limitations

As this was a pragmatic RCT, some participants were enrolled who were not ultimately diagnosed with CAP. However, this reflects the practical difficulties when trying to diagnose and treat a critically ill patient population and was felt to be a relative strength of the trial. The participants included in the study were assessed by treating providers and felt to warrant both vancomycin and respiratory cultures. Most pager alerts were received by medical residents or advanced practice providers, which may have led to delays in action until the results were presented to an attending physician. Additionally, the study did not assess the appropriateness of vancomycin use for non-MRSA indications, which may account for some of the vancomycin used.

Another weakness in the study was the failure of some negative MRSA PCR nasal swab test results to trigger pager alerts. For the 7 patients in whom pager alerts were indicated but did not receive them, no explanation could be determined for why pages were not sent.

The ordering of MRSA PCR nasal swab testing by clinical teams in the control group may have also contributed to the lack of an observed difference in vancomycin-free hours alive between groups. It is possible that participants who were likely to benefit from MRSA PCR nasal swab testing received tests that influenced the results in the control arm. However, analysis of the modified study population did not change the difference in vancomycin-free time, suggesting this “contamination” does not drive the overall results.

## CONCLUSIONS

Methicillin-resistant *Staphylococcus aureus* PCR nasal swab testing demonstrated a high NPV among an ICU population, although its utility as a broad antimicrobial stewardship intervention may be limited in settings with short durations of empiric vancomycin. The effectiveness of existing antimicrobial stewardship–guided de-escalation of empiric anti-MRSA antibiotics should be considered when deciding whether to broadly implement the use of MRSA PCR testing. While it may still be a cost-effective way to reduce vancomycin duration in some ICUs, efforts designed to prevent the initiation of unnecessary anti-MRSA antibiotics in the first place may be more important in many settings.

## Supplementary Material

ciag295_Supplementary_Data

## Supplementary Data

Supplementary materials are available at *Clinical Infectious Diseases* online. Consisting of data provided by the authors to benefit the reader, the posted materials are not copyedited and are the sole responsibility of the authors, so questions or comments should be addressed to the corresponding author.
