# Supplementary material for: Swab Testing to Optimize Pneumonia Treatment With Empiric Vancomycin: A Randomized Controlled Trial
Source: Clin Infect Dis. 2026 Jun 2;83(2):e260–9. doi: 10.1093/cid/ciag295 (PMC13537149; doi:10.1093/cid/ciag295)
Supplement: Supplementary file 1 [file ciag295_supplementary_data.zip › STOP-Vanc Outcomes Manuscript_SUPPLEMENTAL FIGURES_v5.docx]

**Supplement 2**

**Figure S1. Timing of MRSA PCR Nasal Swab Test Order Relative to Study Enrollment.**

**Figure S2. Proportion of Patients Receiving Each Aspect of the Intervention in the Intervention Arm.**

**Figure S3. Proportion of Participants Occupying a Given State Over Time Based on Treatment Arm in Modified Study Population.**

**Figure S4. Distribution of Time Alive Off Vancomycin.**

**Figure S5. 30-day Survival Curves.**

**Figure S6. Competing Risks Modeling of Hospital and ICU Length of Stay.**

**Figure S7.** **Proportion of Participants Occupying a Given Antibiotic State Over Time Based on Treatment Arm.**

**Figure S8. Time from MRSA PCR Nasal Swab Test Result to Discontinuation of Vancomycin Order**

**Table S1. Differential Treatment Effects.**

**Table S2. Concordance of MRSA PCR Nasal Swab Testing with Culture Results**

**Table S3. Proportion of Participants with Infection-related Encounter Diagnoses**

**Table S4. Baseline Demographics and Admission Characteristics for Participants with Encounter Diagnoses Consistent with Bacterial Pneumonia**

**Table S5. Study Outcomes for Participants with Encounter Diagnoses Consistent with Bacterial Pneumonia**

**Figure S1. Timing of MRSA PCR Nasal Swab Test Order Relative to Study Enrollment.** The time at which the earliest MRSA PCR nasal swab test order was placed relative to the timing of study enrollment is shown for all 119 participants in the intervention arm and the 31 participants in the control arm who had testing ordered either prior to study enrollment or in the 7 days after enrollment.

**Figure S2. Proportion of Patients Receiving Each Aspect of the Intervention in the Intervention Arm.** The number of patients who received each step of the study intervention are shown. The percentages reflect the proportion out of the total 119 patients in the intervention arm.

**Figure S3. Proportion of Participants Occupying a Given State Over Time Based on Treatment Arm in Modified Study Population.** The relative proportions of the total number of subjects are displayed over cumulative time post-enrollment as patients occupying one of three states: 1) alive and not on vancomycin (blue), 2) alive and on vancomycin (dark green), or 3) deceased (light green).

**Figure S4. Distribution of Time Alive Off Vancomycin.** Time alive off vancomycin over the first 168 hours (7 days) following study enrollment is shown for study participants in (A) the full study population and (B) the modified study population. Patients who died within the first 168 hours following enrollment are represented with red circles on the plots. Shaded area shows the density distribution of the data for each arm.

**Figure S5. 30-day Survival Curves.** Kaplan-Meier Survival Curves for the Full Study Population (A) and the Modified Study Population (B) are shown along with the differences in the Kaplan-Meier estimates with approximate confidence bands for the differences for the Full Study Population (C) and the Modified Study Population (D). The shaded area's height is one-half of the confidence interval for the difference in the two survival curves, and is drawn such that this area touches the two Kaplan-Meier curves at a time point if and only if there the p-value for comparing those points > 0.05.

**Figure S6. Competing Risks Modeling of Hospital and ICU Length of Stay.** For total hospital length of stay data, the competing probability of hospital discharge alive versus dead are shown for the Full Study Population (A) and the Modified Study Population (B). For ICU length of stay data, the competing probability of ICU discharge alive versus dead are shown for the Full Study Population (C) and the Modified Study Population (D). The dashed lines indicate the probability of patients being discharged alive from either the hospital (A & C) or from the ICU (B & D) over time while the solid lines indicate the probability of patients passing away prior to discharge. Probabilities for patients in the control group are shown in blue while probabilities for the intervention group are shown in green.

**Figure S7.** **Proportion of Participants Occupying a Given Antibiotic State Over Time Based on Treatment Arm.** The proportion of participants in the Full Study Population occupying a given antibiotic state are shown for IV antibiotics (A) and all antibiotics (C). The proportion of participants in the Modified Study Population occupying a given antibiotic state are shown for IV antibiotics (B) and all antibiotics (D), as well.

**Figure S8. Time from MRSA PCR Nasal Swab Test Result to Discontinuation of Vancomycin Order.** Kaplan-Meier Curves showing the time from the availability of the MRSA PCR nasal swab test result in the EHR to the discontinuation of the order for vancomycin for the Full Study Population (A) and the Modified Study Population (B) over the first 7 days (168 hours) following the release of the test result.

**Table S1. Differential Treatment Effects.**

| A | Full Study Population |  |  |
| --- | --- | --- | --- |
| Variable | | χ^2^ | *P* value |
|  | |  |  |
| Age | | 1.19 | 0.275 |
| SOFA score | | 0.014 | 0.908 |
| Baseline Creatinine | | 0.76 | 0.382 |
| Immunocompromised Status | | 2.06 | 0.152 |
| All Covariates | | 3.47 | 0.483 |
|  | |  |  |
| χ^2^- partial likelihood ratio χ² statistic | |  |  |
|  |  |  |  |
| B | Modified Study Population |  |  |
| Variable | | χ^2^ | *P* value |
|  | |  |  |
| Age | | 0.0019 | 0.966 |
| SOFA score | | 0.56 | 0.452 |
| Baseline Creatinine | | 1.18 | 0.277 |
| Immunocompromised Status | | 1.20 | 0.273 |
| All Covariates | | 2.36 | 0.670 |

χ^2^- partial likelihood ratio χ² statistic

| **Table S2. Concordance of MRSA PCR Nasal Swab Testing with Culture Results** | | | | | | | | |  |
| --- | --- | --- | --- | --- | --- | --- | --- | --- | --- |
|  | | **No. (%)** | | | | | | | |
|  |  | **MRSA PCR Nasal Swab Test Results and  Respiratory Culture Results** | |  | **MRSA PCR Nasal Swab Test Results and  Blood Culture Results** | |  | **MRSA PCR Nasal Swab Test Results and  Blood or Respiratory Culture Results** | |
| **Measure** | | **Intervention (n = 71)** | **Control (n = 18)** |  | **Intervention (n = 112)** | **Control (n = 29)** |  | **Intervention (n = 115)** | **Control (n = 30)** |
| Subjects with MRSA in culture^a^ | | 5 (8) | 0 (0) |  | 3 (3) | 0 (0) |  | 8 (7) | 0 (0) |
| MRSA PCR Nasal Swab Test (-) | | 56 (79) | 17 (94) |  | 91 (81) | 27 (93) |  | 94 (82) | 28 (93) |
|  | MRSA in culture^a^ | 0/56 (0) | 0/17 (0) |  | 1/91 (1) | 0/27 (0) |  | 1/94 (1) | 0/28 (0) |
| MRSA PCR Nasal Swab Test (+) | | 15 (21) | 1 (6) |  | 21 (19) | 2 (7) |  | 21 (18) | 2 (7) |
|  | MRSA in culture^a^ | 5/15 (33) | 0/1 (0) |  | 2/21 (10) | 0/2 (0) |  | 7/21 (33) | 0/2 (0) |
|  |  |  |  |  |  |  |  |  |  |
| **Test Performance^b^** | |  |  |  |  |  |  |  |  |
| Sensitivity (95% CI) | | 1.000 (0.566 to 1.000)  0.869 (0.781 to 0.925)  0.313 (0.142 to 0.556)  1.000 (0.950 to 1.000) | |  | 0.667 (0.119 to 0.983)  0.848 (0.779 to 0.898)  0.087 (0.015 to 0.268)  0.992 (0.954 to 1.000) | |  | 0.875 (0.529 to 0.994)  0.883 (0.819 to 0.927)  0.304 (0.156 to 0.509)  0.992 (0.955 to 1.000) | |
| Specificity (95% CI) | |  |  |  |  |  |  |  |  |
| PPV (95% CI) | |  |  |  |  |  |  |  |  |
| NPV (95% CI) | |  |  |  |  |  |  |  |  |
| ^a^ MRSA was considered to be present if, in the given culture type there was MRSA recovered from a culture collected either prior to enrollment or within the first 7 days following enrollment.  ^b^The presence of MRSA in cultures as defined above was used as a reference for determining test performance. Subjects were excluded from the analysis if no cultures with results existed. Performance of swabs from both control and intervention arm are included together in this table. | | | | | | | | | |

| **Table S3. Proportion of Participants with Final Encounter Diagnoses Associated with Infections** | | | |  |
| --- | --- | --- | --- | --- |
|  | |  |  | |
|  |  | **No. (%)** | |  |
| **Encounter Diagnosis** | | **Intervention (n = 119)** | **Control (n = 158)** | |
| Associated with bacterial pneumonia^a^ | | 58 (49) | 75 (48) | |
| Associated with pneumonia, only viral^b^ | | 11 (9) | 9 (6) | |
| Associated with bacterial infection other than pneumonia^c^ | | 33 (28) | 49 (31) | |
| Encounter diagnoses other than pneumonia or bacterial infections^d^ | | 19 (16) | 26 (17) | |
|  |  |  |  | |
| ^a^Includes participants either with an ICD10 encounter diagnosis code associated specifically with bacterial pneumonia, an ICD10 encounter diagnosis code associated with pneumonia and no code for a viral infection, or an ICD10 encounter diagnosis code associated with either pneumonia or sepsis and a respiratory culture (collected either prior to enrollment or in the 7 days following enrollment) with a bacterial pathogen identified. | | | |  |
| ^b^Includes participants with an ICD10 encounter diagnosis code associated specifically with viral pneumonia, and not meeting criteria for inclusion in the "Associated with bacterial pneumonia" group | | | |  |
| ^c^Includes participants who do not meet criteria for inclusion in the "Associated with bacterial pneumonia" group and have either an ICD10 encounter diagnosis code associated specifically with a bacterial infection or an ICD10 encounter code associated with sepsis and no code for a viral infection. | | | |  |
| ^d^Includes all participants who do not meet the criteria for inclusion in the "Associated with bacterial pneumonia" group, the "Associated with pneumonia, only viral" group, or the "Associated with bacterial Infection other than pneumonia" group | | | |  |

**Table S4. Baseline Demographics and Admission Characteristics for Participants with Encounter Diagnoses Associated with Bacterial Pneumonia**

|  |  | **No. (%)** |  |  |
| --- | --- | --- | --- | --- |
|  |  | **Population with Encounter Diagnoses Associated with Bacterial Pneumonia** | | |
| **Characteristic** | | **All (n = 133)** | **Intervention (n = 58)** | **Control (n = 75)** |
| **Participant Demographics** | |  |  |  |
| Age, median (IQR), y | | 61 (48-69) | 61 (49-68) | 63 (46-71) |
| Sex |  |  |  |  |
|  | Female | 43 (32) | 20 (34) | 23 (31) |
|  | Male | 90 (68) | 38 (66) | 52 (69) |
| Race^a^ | |  |  |  |
|  | White | 110 (83) | 50 (86) | 60 (80) |
|  | Black/African American | 20 (15) | 7 (12) | 13 (17) |
|  | Other | 2 (2) | 1 (2) | 1 (1) |
|  | Unknown | 7 (5) | 2 (3) | 5 (7) |
| Ethnicity | |  |  |  |
|  | Hispanic | 4 (3) | 3 (5) | 1 (1) |
|  | Non-Hispanic | 121 (91) | 152 (90) | 69 (92) |
|  | Unknown | 8 (6) | 3 (5) | 5 (7) |
| Elixhauser Comorbidity Index, median (IQR) | | 14 (4-24) | 13 (3-23) | 16 (6-25) |
| Baseline CKD present | | 25 (19) | 11 (19) | 14 (19) |
|  | Baseline ESRD | 4 (3) | 1 (2) | 3 (4) |
| Immunocompromised | | 18 (14) | 8 (14) | 10 (13) |
|  | HIV | 5 (4) | 2 (3) | 3 (4) |
|  | Solid Organ Transplant | 11 (8) | 5 (9) | 6 (8) |
|  | Stem Cell Transplant | 2 (2) | 1 (2) | 1 (1) |
| Body Mass Index, mean (SD) kg/m^2^ | | 27 (8) | 27 (7) | 26 (8) |
| **Admission Characteristics** | |  |  |  |
| SOFA score on admission to ICU, median (IQR) | | 7 (5-11) | 6 (4-10) | 8 (5-11) |
| Requiring Supplemental Oxygen | | 121 (91) | 52 (90) | 69 (92) |
| Requiring Mechanical Ventilation | | 41 (31) | 18 (31) | 23 (31) |
| Requiring Vasopressor Support | | 61 (46) | 23 (40) | 38 (51) |
| Reference Creatinine, median (IQR) mg/dL^b^ | | 1.2 (0.8-2.1) | 1.2 (0.9-2.2) | 1.2 (0.8-2.0) |
| Blood or respiratory cultures collected | | 132 (99) | 57 (98) | 75 (100) |
|  | Number of blood or respiratory cultures collected, median (IQR) | 3 (2-4) | 3 (2-4) | 3 (2-4) |
| Blood cultures collected | | 125 (94) | 55 (95) | 70 (93) |
|  | Number of blood cultures collected, median (IQR) | 2 (2-4) | 2 (2-4) | 2 (2-3) |
| Respiratory cultures collected | | 97 (73) | 40 (69) | 57 (76) |
|  | Number of respiratory cultures collected, median (IQR) | 1 (0-1) | 1 (0-1) | 1 (0-1) |
| MRSA PCR nasal swab result in the previous 30 days^c^ | | 8 (6) | 5 (9) | 3 (4) |
| Blood or Respiratory Cultures with MRSA^d^ | | 8 (6) | 4 (7) | 4 (5) |
| ^a^Percentages do not necessarily equal 100% as race categories were not mutually exclusive | | | | |
| ^b^Reference creatinine was defined as the most recent creatinine obtained since admission but prior to the administration of the first dose of vancomycin. If no such value existed than the most immediate creatinine following the administration of vancomycin was used.  ^c^Includes MRSA PCR nasal swabs if they were ordered in the 30 days prior to enrollment, if they occurred in a separate hospital admission from the study admission.  ^d^Among cultures collected either prior to enrollment or within the first 7 days following enrollment | | | | |
|  | | | |  |

**Table S5. Study Outcomes for Participants with Encounter Diagnoses Associated with Bacterial Pneumonia**

|  |  | **No. (%)** | |  |  |  |
| --- | --- | --- | --- | --- | --- | --- |
| **Outcome** | | **Intervention (n = 58)** | **Control (n = 75)** | **Difference (95% CI)** | **Odds Ratio (95% CI)** | ***P* value** |
| Primary outcome | |  |  |  |  |  |
|  | Treatment transition odds ratio^a^ |  |  |  | 1.031 (0.735 to 1.421) | 0.812 |
|  | Estimated expected number of vancomycin-free hours alive, h^b,c^ | 103.5 | 101.6 | 1.9 (-17.5 to 20.8) |  | 0.812 |
|  | Estimated expected number of hours receiving vancomycin, h^b,c^ | 43.5 | 44.4 | -0.8 (-8.4 to 4.9) |  |  |
|  | Estimated expected number of hours alive, h^b,c^ | 147.0 | 146.0 | 1.1 (-11.3 to 11.7) |  |  |
| Secondary outcomes | |  |  |  |  |  |
|  | Time alive off vancomycin, median (IQR), h^b,d^ | 134 (106-159) | 120 (55-148) | 18.9 (0.6 to 37.2) |  | 0.049 |
|  | 30-day all-cause mortality | 13 (22) | 31 (41) |  | 0.476 (0.213 to 1.065) | 0.035 |
| Exploratory outcomes | |  |  |  |  |  |
|  | AKI^e^ | 15 (26) | 25 (35) |  | 0.759 (0.342 to 1.685) | 0.405 |
|  | In-hospital all-cause mortality | 10 (17) | 26 (35) |  | 0.432 (0.181 to 1.032) | 0.041 |
|  | Mean length of stay, d | 12.6 | 10.8 |  | 1.043 (0.698 to 1.558) | 0.839 |
|  | Mean ICU length of stay, d | 5.9 | 5.8 |  | 1.173 (0.797 to 1.726) | 0.418 |
|  | Mean IV antibiotic-free days alive, d^f^ | 6.26 | 5.23 | 1.02 (-0.80 to 2.56) | 1.279 (0.808 to 1.860) | 0.140 |
|  | Mean time on IV antibiotics, d^f^ | 5.64 | 6.04 | -0.40 (-1.01 to 0.44) | 1.279 (0.829 to 1.832) |  |
|  | Mean antibiotic-free days alive, d^f^ | 4.45 | 3.53 | 0.92 (-0.60 to 2.29) | 1.280 (0.835 to 1.858) | 0.158 |
|  | Mean time on antibiotics, d^f^ | 7.29 | 7.59 | -0.30 (-0.81 to 0.27) | 1.280 (0.866 to 1.897) |  |
|  | Mean Ventilator-free days, d^f^ | 11.08 | 9.22 |  | 2.136 (1.078 to 4.233) | 0.030 |
|  | Receipt of alternative anti-MRSA antibiotics^g^ | 3 (5) | 3 (4) |  | 1.001 (0.166 to 6.025) | 1.000 |

^a^Odds ratio of >1 indicates higher odds of transitioning to a “worse” state in the control group in the three-state model with alive and not on vancomycin being the best state and deceased being the worst state

^b^Value out of the first 168 hours (7 days) following enrollment

^c^Estimated expected number of hours based on a patient’s odds of transitioning from their current state to a worse state using a three-state proportional-odds state transition model. Estimated expected values denote model-based estimates of the mean [26]

^d^Median number of actual hours alive and free of vancomycin

^e^Defined as an increase in creatinine to ≥ 1.5 times the reference creatinine level or an increase by ≥ 0.3 mg/dL within 14 days of study enrollment. Excludes participants who have end stage renal disease (ESRD) prior to study enrollment

^f^Value out of the first 14 days following enrollment

^g^Includes any antibiotics considered to be appropriate options for the treatment of MRSA pneumonia (as pre-specified in the study protocol), regardless of the actual reason for their use during the study
