# Supplementary material for: Swab Testing to Optimize Pneumonia Treatment With Empiric Vancomycin: A Randomized Controlled Trial
Source: Clin Infect Dis. 2026 Jun 2;83(2):e260–9. doi: 10.1093/cid/ciag295 (PMC13537149; doi:10.1093/cid/ciag295)
Supplement: Supplementary file 1 [file ciag295_supplementary_data.zip › STOP-Vanc_SUPPLEMENT_1_BINDER.pdf]

Supplement 1

Study Protocol and Statistical Analysis Plan

Trial: Swab Testing to Optimize Pneumonia treatment with empiric Vancomycin (STOP-Vanc)

Manuscript: Swab Testing to Optimize Pneumonia treatment with empiric Vancomycin (STOP-Vanc): A Randomized Controlled Trial

ClinicalTrials.gov: NCT06272994

Authors: Jeffrey A. Freiberg; Edward T. Qian; Kylie G. Nairon; Benjamin J. Ereshefsky; Cassandra Hennessy; Grace Van Winkle; Ariel A. Lewis; Joy M. Justice; Justin K. Siemann; Joanna L. Stollings; Taylor M. Rali; Frank E. Harrell; Cheryl L. Gatto; George E. Nelson; Todd W. Rice for the Vanderbilt Center for Learning Healthcare

This Supplement contains the following items:

1) Final Protocol (dated 10/29/2024)
2) Summary of changes to Study Protocol from initial to final version
3) Final Statistical Analysis Plan (dated 1/27/2025)
4) Summary of changes to the Statistical Analysis Plan from initial to final version

Swab Testing to Optimize Pneumonia treatment with empiric Vancomycin (STOP-Vanc)

Study Investigators:
Jeffrey Freiberg, MD, PhD, Instructor in Medicine, Department of Medicine, Division of Infectious Diseases

Benjamin Ereshefsky, PharmD, BCIDP, Infectious Diseases Pharmacist, Department of Pharmaceutical Services

Cheryl L. Gatto, PhD, PMP, Research Associate Professor, Department of Emergency Medicine, Director Vanderbilt Institute for Clinical and Translational Research

Frank E. Harrell Jr., PhD, Professor, Department of Biostatistics

Cassandra Hennessy, MS, Senior Biostatistician, Department of Biostatistics

Kylie Nairon, PhD, Scientific Research Project Manager, VICTR

George Nelson, MD, Associate Professor, Department of Medicine, Division of Infectious Diseases

Edward Qian, MD, Assistant Professor, Department of Medicine, Division of Allergy, Pulmonary, and Critical Care

Todd Rice, MD, Professor, Department of Medicine, Division of Allergy, Pulmonary, and Critical Care

History of Protocol Amendments

Version	Submission Date	Changes Made
1	10/8/2023	N/A
2	2/1/2024	- Study title changed to "Swab Testing to Optimize Pneumonia treatment with empiric Vancomycin (STOP-Vanc)
3	7/16/2024	- Study coordinator changed from Justin Siemann to Kylie Nairon
4	8/22/24	- Maximum sample size increased to 260 with rationale, study coordinator information added to title page
5	10/29/24	- Maximum sample size increased to 300 with rationale

Table of Contents:

1.0 Study Summary ... 5
2.0 Background ... 6
3.0 Rational and Specific Aims ... 7
4.0 Study Design ... 8
5.0 Inclusion/Exclusion Criteria ... 9
5.1 Inclusion criteria ... 9
5.2 Exclusion criteria ... 9
6.0 Enrollment/Randomization ... 10
6.1 Study Sites ... 10
6.2 Study Population ... 10
6.3 Enrollment ... 10
6.4 Consent ... 10
6.5 Randomization ... 11
7.0 Study Procedures ... 12
7.1 EMR based screening for eligible patients ... 12
7.2 MRSA nasal swab PCR testing ... 12
7.3 EMR based antimicrobial stewardship program guidance ... 12
7.4 Pharmacist-reinforced education ... 12
7.5 Blinding ... 13
8.0 Data Collection ... 14
9.0 Risks and Benefits ... 18
9.1 Risks ... 18
9.2 Benefits ... 18
10.0 Reporting of Adverse Events or Unanticipated Problems ... 19
11.0 Study Withdrawal/Discontinuation ... 20
12.0 Statistical Considerations ... 21
12.1 Statistical analysis ... 21
12.2 Sample size calculations ... 21
12.3 Baseline Characteristics ... 21
12.4 Primary Outcome ... 22
12.5 Secondary Outcomes ... 22
12.6 Exploratory Outcomes ... 22

13.0 Privacy/Confidentiality Issues 25
14.0 Follow-up and Record Retention 26
15.0 References 27

1.0 Study Summary

Title	Swab Testing to Optimize Pneumonia treatment with empiric Vancomycin (STOP-Vanc)
Methodology	Single center, pragmatic, randomized clinical trial (pRCT)
Study Center	Vanderbilt University Medical Center (VUMC)
Primary Aim	To examine whether reporting the results of a negative rapid PCR back to the provider via a pager alert results in decreased vancomycin utilization for critically ill adults with community-acquired pneumonia when compared with usual care.
Inclusion and Exclusion Criteria	Inclusion criteria<br>- Adult (age greater than or equal to 18) patients admitted/transferred to the Vanderbilt University Medical Center (VUMC) Medical Intensive Care Unit (MICU) from the VUMC Emergency Department or from a hospital floor within 48 hours of admission.<br>- Suspicion for CAP (defined as an indication for antibiotics of a “respiratory infection” and/or an order for a respiratory culture i.e., sputum culture, tracheal aspirate culture, or bronchoalveolar lavage (BAL) culture).<br>- No topical nasal decolonization during hospitalization prior to collection of MRSA nasal swab PCR.<br>- Must match both of the following in either order:<br>o The patient has been admitted to and physically located in the MICU.<br>o The patient has received a continuing vancomycin order, or a pharmacokinetics consult for a continuing vancomycin order, no later than 24 hours following their physical admission to the MICU.<br><br>Exclusion criteria<br>- Hospital stay of longer than 48 hours prior to MICU admission.<br>- Known to be a prisoner
Study Intervention	Patients eligible for this study will then be randomized in a 1:1 ratio for the results of the MRSA nasal swab PCR to be transmitted through the electronic health record (EHR) to the treating clinicians (intervention group), or not (control group). For the patients assigned to the intervention group who have a negative MRSA nasal swab PCR result, a provider pager alert will direct clinicians to clinical guidance recommending discontinuing vancomycin, if clinically appropriate.
Primary Outcome	Vancomycin-free hours alive, defined as the number of hours alive and free of the use of vancomycin within the first seven days following enrollment in the trial.
Secondary Outcomes	30-day all-cause mortality<br>Time Alive off Vancomycin

2.0 Background

Methicillin-resistant *Staphylococcus aureus* (MRSA) is a critical antimicrobial resistant threat responsible for greater than 300,000 inpatient infections and 15,000 deaths per year in the United States (1, 2). Community-acquired pneumonia (CAP) is a major driver of hospital antibiotic use. Nationally, there are around 600,000 CAP-related hospital admissions annually. However, MRSA is an infrequent cause of community-acquired pneumonia (CAP), accounting for less than 1% of cases (3). Despite this, MRSA is a frequently feared cause of CAP, which leads to the frequent use of vancomycin, an anti-MRSA antibiotic, in empiric CAP treatment.

At VUMC, vancomycin is the most commonly used antimicrobial overall. A VUMC vancomycin medication use evaluation (MUE) of >11,000 hospitalizations found that if stopped after three days without a positive culture, VUH empiric vancomycin use would decrease by 61%. There is disproportionately high vancomycin use compared to the prevalence of MRSA at VUMC (2.7% in the ICU, 0.7% overall) (3).

Inappropriate antibiotic use can lead to avoidable adverse drug events and costs, as well as drive antimicrobial resistance. Empiric vancomycin use in patients hospitalized for pneumonia has demonstrated increased mortality, acute kidney injury (AKI), and secondary infections (4). The use of vancomycin is unfortunately associated with a high risk for toxicity and serious adverse events. Up to two-thirds of patients receiving high dose vancomycin develop AKI (5, 6). Additionally, bone marrow suppression, linear IgA bullous dermatosis, anaphylaxis, and life-threatening hypersensitivity reactions are seen with vancomycin use (7-10). Furthermore, vancomycin is a costly antibiotic to use in the hospital as it requires careful monitoring due to its narrow therapeutic range and high risk of toxicity (11). Annually, VUMC spends more than $610,000 on vancomycin, about 10% of total antimicrobial expenditures, making it the single most costly antimicrobial. Therefore, reducing inappropriate use of vancomycin in patients with CAP has the potential to improve patient outcomes while at the same time providing overall cost savings.

There is growing data to support the use of MRSA nasal swabs as a screening method to guide de-escalation of vancomycin use in CAP. A 2018 meta-analysis found using nasal swabs for MRSA screening had an overall 96.5% negative predictive value (NPV) for pneumonia, which was increased to 98.1% among patients with CAP or Healthcare-associated pneumonia (HCAP) (12). Multiple retrospective studies along with one prospective study utilizing MRSA nasal swab-based de-escalation protocols have shown MRSA nasal swab use to be effective in decreasing vancomycin use and associated costs without having any negative effects on patient outcomes (13-21). Among these studies, significant decreases in hospital length of stay (13) and rate of AKI (19) have been shown. Furthermore, the use of MRSA detection in nasal swabs is now consistent with guideline-based management of CAP (22). However, all the aforementioned studies are quasi-experimental analyses. To date there are no randomized controlled studies of the use of MRSA nasal swab guided antibiotic de-escalation.

3.0 Rational and Specific Aims

Our primary aim is to determine the effect of implementing MRSA PCR nasal swabs with rapid return of results via a pager alert which will inform the physicians of the result and direct them to clinical guidance on the use of vancomycin in cases of community-acquired pneumonia. We hypothesize that implementation of this screening in conjunction with antimicrobial stewardship guidance on interpretation of the results will lead to reduced use of vancomycin. Furthermore, as secondary aims we anticipate a reduction in vancomycin-associated adverse events and an overall cost-savings, all without increasing mortality or hospital readmissions.

4.0 Study Design

This is a single center, pragmatic, randomized clinical trial (pRCT) examining whether reporting the results of a negative rapid PCR back to the provider via a pager alert results in decreased vancomycin utilization for critically ill adults with community-acquired pneumonia when compared with usual care. Patients eligible for this study and randomized to the intervention arm, will have a nasal swab collected and sent to the clinical laboratory for the MRSA nasal swab PCR test to be run. Eligible patients who are randomized to the control arm will not have a nasal swab collected. Patients eligible for this study will be randomized in a 1:1 ratio for the results of the MRSA nasal swab PCR transmitted through the electronic health record (EHR) to the treating clinicians (intervention group). For the patients assigned to the intervention group who have a negative MRSA nasal swab PCR result, providers will receive a pager alert which inform them of the negative result and will direct clinicians to clinical guidance recommending clinicians to discontinue vancomycin, if clinically appropriate. Intervention will solely involve providing the aforementioned test result along with antimicrobial stewardship guidance via the Vanderbilt Antimicrobial Stewardship Program (VASP) CAP guidelines. The decisions regarding ordering or discontinuation of antibiotics will be ultimately at the discretion of the treating provider. Data will be collected prospectively from the medical record to determine the effect of the assigned intervention on outcomes.

5.0 Inclusion/Exclusion Criteria

5.1 Inclusion criteria

- Adult (age greater than or equal to 18) patients admitted/transferred to the Vanderbilt University Medical Center (VUMC) Medical Intensive Care Unit (MICU) from the VUMC Emergency Department or from a hospital floor within 48 hours of admission.
- Suspicion for pneumonia on admission (defined as an indication for antibiotics of "respiratory infection" and/or an order for a respiratory culture i.e., sputum culture, tracheal aspirate culture, or bronchoalveolar lavage (BAL) culture).
- No topical nasal decolonization during hospitalization prior to collection of MRSA nasal swab PCR.
- Must match both of the following in either order:
  - The patient has been admitted to and physically located in the MICU.
  - The patient has received a continuing vancomycin order, or a pharmacokinetics consult for a continuing vancomycin order, no later than 24 hours following their physical admission to the MICU.

5.2 Exclusion criteria

- Hospital stay of longer than 48 hours prior to MICU admission.
- Known to be a prisoner

6.0 Enrollment/Randomization

6.1 Study Sites

This study will be conducted solely in the VUMC MICU.

6.2 Study Population

Adult (age greater than or equal to 18) patients admitted/transferred to the VUMC Medical Intensive Care Unit who are receiving vancomycin for CAP. All eligible patients will be included and there will be no selection based on gender, race, weight, or other clinical factors.

6.3 Enrollment

Patients will automatically be considered for enrollment either upon transfer to the MICU with an order for "pharmacy consult for vancomycin dosing" already in place, or if an order for vancomycin and/or a "pharmacy consult for vancomycin dosing" order is placed (within 24 hours of admission to unit) for a patient located in the MICU, who has not received any topical nasal decolonization during the hospitalization prior to collection of MRSA nasal swab PCR. The reasons for ineligibility of patients screened but not enrolled will be recorded.

6.4 Consent

The decision to initiate and continue empiric vancomycin is currently done at the discretion of the patient's provider, who takes into account a multitude of factors including the patient's clinical status, history, and available test results. While some observational studies suggest the use of a rapid PCR-based MRSA nasal swabs improve outcomes beyond just improving antimicrobial stewardship, this has not yet been examined by a RCT. As there is very little potential harm in obtaining a nasal swab, and the intervention in this trial would solely be to provide clinical guidance, we feel that there is minimal risk involved in this study. The use of a nasal swab to test for MRSA is minimal risk as a nasal swab is not considered an invasive test. Patients in the ICU receive twice daily nasal swabs for nasal decolonization while in the ICU as part of routine clinical practice. The risk to patients from the nasal swabs to detect MRSA is less than the risk from the routine nasal decolonization swabs as the swabs for MRSA detection do not contain any drugs. These routine nasal decolonization swabs are done as part of regular care without written consent from patients. Patients will have the same rights to refuse nasal MRSA swabs similar to their ability to refuse the routine nasal decolonization. In addition, this study is also minimal risk because it does not make any decisions regarding the treatments offered to patients. Instead, it only provides information and antimicrobial stewardship guidance to patient's providers who retain complete authority over what is the appropriate care for their patients.

In addition to the minimal risk posed by the study, obtaining informed consent prior to enrollment in this study would not be feasible or practicable for multiple reasons:

1) The potential benefit of MRSA screening is dependent on early, rapid testing to generate results that are readily available to help guide the providers' decisions. This means that the swab must be ordered immediately at the time that the ICU provider decides to start or continue antibiotics, and prior to routine decolonization that is performed in all patients admitted to the ICUs at Vanderbilt University Medical Center. This event typically occurs simultaneously with a patient's arrival to the ICU. If the decision to obtain a MRSA nares swab is delayed until written consent is acquired, it would be too late to study its effect.
2) The MRSA nasal swabs are only considered valid if they are collected prior to nasal decolonization. Since nasal decolonization with the use of nasal swabs occurs regularly twice a day in all ICU patients, at VUMC, and most critically ill patients are temporarily cognitively impaired due to their critical illness, it would not be possible to obtain written consent prior to decolonization in most cases.
3) The goal of the study is to test whether the use of a MRSA nares swab would reduce the length of time that patients receive unnecessary antibiotics, it must be done at the same time as the decision to start or continue antibiotics is made. Since the use of antibiotics in the ICU patient is an urgent intervention in many patients, it would be inappropriate to delay the receipt of antibiotics in some of the most critically ill patients in order to get written consent.
4) The decision to initiate antibiotics is often made when patients are critically ill and unable to consent for themselves, therefore it would often not be feasible to obtain consent from a patient or their representative at the time at which the MRSA nasal swab would need to be performed.
5) Consenting the participants may prompt either the clinical team or the participant to order (or ask for) the MRSA nasal swab in patients randomized to the control arm (and thus, randomized not to receive the nasal swab prior to decolonization). This would potentially lead the clinical team to order the MRSA nasal swab prior to decolonization in the control arm as well, which would not allow the study team to truly evaluate the role of an admission nasal swab in reducing vancomycin use. This contamination of the control arm would bias the study results to not being able to see the effect of the use of these MRSA nasal swabs prior to decolonization on vancomycin use.

For all of these reasons, this study could not be done practicably without a waiver of consent, and it would be impractical to obtain written informed consent. Therefore, given that this study presents minimal risk, it would not adversely affect the welfare or privacy rights of the participant, and it would be impracticable to obtain consent, we will request a waiver of informed consent.

6.5 Randomization

Eligible patients will be automatically enrolled in this study and randomized in a 1:1 fashion to either the control or the study group.

7.0 Study Procedures

7.1 EMR based screening for eligible patients

When an order for vancomycin or a consult for "pharmacy to dose vancomycin" is placed for a patient located in the MICU, or a patient is transferred to the MICU with an order for "pharmacy to dose vancomycin" already present, a software application in the electronic medical record (EMR) will automatically assess a patient's eligibility for this study based on whether they meet the inclusion criteria and assuming they do not meet any of the exclusion criteria.

7.2 MRSA nasal swab PCR testing

For patients who meet the study criteria, as outlined above, and are randomized to the intervention arm of the study, a MRSA nasal swab will be collected on admission to the MICU prior to topical nasal decolonization. Then a PCR test will be ordered to be run on the collected MRSA nasal swab (no order will be placed for PCR testing and no nasal swabs will be collected on patients enrolled in the control arm). Patients will be randomized in a 1:1 fashion on an individual patient level. For patient's randomized to the intervention group, once the MRSA nasal swab PCR has been completed, the results will be available in the EMR along with antimicrobial stewardship program guidance as outlined below.

7.3 Pager/EMR based antimicrobial stewardship program guidance

For patients in the study group with a negative MRSA nasal swab PCR, a pager alert will be sent to the patient's primary team. The pager alert will inform the provider of the negative test result and will direct them to the VASP CAP guidelines where they will receive further clinical guidance recommending the provider consider discontinuing vancomycin.

A sample pager alert text is included below:

"FIRST/MIDDLE/LAST INITIALS ROOM #: MRSA NASAL PCR is NEGATIVE, the presence of MRSA pneumonia is UNLIKELY. If vancomycin is for empiric pneumonia treatment, consider DISCONTINUING VANCOMYCIN. See VASP website: bit.ly/VUMC-CAP for more information."

7.4 Pharmacist-reinforced education

In addition to the direct, instantaneous antimicrobial stewardship guidance to be delivered through a pager alert as outlined above, general education about the use of MRSA nasal swab PCR testing to guide de-escalation of empiric vancomycin will

be provided by the MICU pharmacist when a patient with a negative MRSA nasal swab PCR is continued on vancomycin.

7.5 Blinding

Given that this study relies on using diagnostic testing to provide guidance to providers, it is not feasible to blind providers in this study. Likewise, patients will not be explicitly blinded as this would not be feasible if the patient's providers must discuss their rationale for discontinuing or continuing antibiotics as part of informed, shared decision making.

8.0 Data Collection

For each study subject in the intervention arm, the following test(s) will be performed:

- MRSA nasal swab PCR

For each study subject in the control arm, the following test(s) will be performed:

- None

For each study subject, the following data may be collected from the EMR:

- Age
- Sex
- Race (allow missingness/multiple)
- Ethnicity (allow missingness/multiple)
- Height (m)/weight (kg)/BMI
- Elixhauser comorbidity index
- Baseline CKD
- ESRD status at time of admission
- Immunocompromised/immunosuppressed status (HIV, solid organ transplant recipient, stem cell transplant recipient)
- Reference Creatinine - Defined as the most recent creatinine obtained since admission but prior to the administration of the first dose of vancomycin. If no value exists than the most immediate creatinine following the administration of vancomycin will be used
- Date/Time of presentation to VUMC
- Date/Time of admission to MICU
- Date/Time of administration of first dose of vancomycin
- Date/Time of administration of last dose of vancomycin
- Date/Time "Pharmacy consult for vancomycin dosing" order placed
- Date/Time "Pharmacy consult for vancomycin dosing" order discontinued
- Date/Time vancomycin order restarted
- Number of doses of vancomycin received within 14 days of enrollment
- Date/Time of vancomycin doses within 14 days of enrollment
- Values of doses of vancomycin received within 14 days of enrollment
- Number of vancomycin levels drawn within 14 days of enrollment
- Date/Time of vancomycin levels within 14 days of enrollment
- Date/Time of topical nasal decolonization
- Date/Time of MRSA nasal swab order placed
- Date/Time of collection of MRSA nasal swab sample (if applicable)
- Date/Time of MRSA nasal swab PCR result (if applicable)
- MRSA nasal swab results (positive or negative) (if applicable)
- Date/Time of transfer out of MICU
- Date/Time of discharge
- Status (alive or dead) on discharge

- Receipt of antibiotics at discharge
- Date/Time of any oral or IV antibiotic doses 48 hours prior to enrollment through hospital discharge or 14 days post-enrollment, whichever is sooner
- Name of any oral or IV antibiotic administered 48 hours prior to enrollment through hospital discharge or 14 days post-enrollment, whichever is sooner
- Route of administration of any oral or IV antibiotic received 48 hours prior to enrollment through hospital discharge or 14 days post-enrollment, whichever is sooner
- Date/Time of any parenteral antibiotic 48 hours prior to enrollment through hospital discharge or 14 days post-enrollment, whichever is sooner
- Name of any parenteral antibiotic administered 48 hours prior to enrollment through hospital discharge or 14 days post-enrollment, whichever is sooner
- Route of administration of any parenteral antibiotic received 48 hours prior to enrollment through hospital discharge or 14 days post-enrollment, whichever is sooner
- Date/Time of any doses of linezolid received 48 hours prior to enrollment through hospital discharge or 14 days post-enrollment, whichever is sooner
- Date/Time of any doses of ceftaroline received 48 hours prior to enrollment through hospital discharge or 14 days post-enrollment, whichever is sooner
- Status (alive or dead) at 30 days
- SOFA score upon ICU admission
- Baseline respiratory support status
- Whether patient required intubation at any point during hospitalization
- Whether patient required vasopressors at any point during hospitalization
- All-cause readmission within 30 days
- Peak creatinine level within 14 days of enrollment
- Respiratory culture (sputum culture, tracheal aspirate culture, or BAL culture) results from cultures collected from admission to VUMC to 7 days after enrollment
- Blood culture results from cultures collected from admission to VUMC to 7 days after enrollment

The following study variables will be calculated:

- Vancomycin-free hours alive
    - The number of hours alive and free of the use of vancomycin within the first 168 hours (seven days) following enrollment in the trial.
- Length of stay (LOS)
    - Difference in days from date of presentation to VUMC to date of discharge, inclusive of first and last dates.
- Total ICU LOS
    - Total Difference in hours from time of admission to MICU to transfer out of MICU (summed if multiple separate transfers to MICU during the same hospitalization).
- Time Alive off Vancomycin

  - The number of hours out of the 168 hours (7 days) following enrollment in the trial that the patient is alive and not receiving vancomycin. Time receiving vancomycin will be defined as the difference from time of administration of the first dose of vancomycin to time of administration of the last dose of vancomycin. If the patient was discharged prior to study day 7 and received vancomycin within 24 hours of discharge then the total duration of vancomycin will be calculated based on the anticipated last day of vancomycin at the time of discharge or else by using study day 7 as an end point, whichever is sooner.
- Total duration of antibiotic exposure
  - Difference in days from time of first dose of any oral or IV antibiotic to the last dose of antibiotics. If the patient was discharged prior to study day 14 and received antibiotics within 24 hours of discharge then the total duration of antibiotics will be calculated based on the anticipated last day of antibiotics at the time of discharge or else by using study day 14 as an end point, whichever is sooner.
- Total duration of parenteral antibiotic exposure
  - Difference in days from time of first dose of any parenteral antibiotic to the last dose of parenteral antibiotics, inclusive of first and last dates. If the patient was discharged prior to study day 14 and had received parenteral antibiotics within 24 hours of discharge then the total duration of parenteral antibiotics will be calculated based on the anticipated last day of parenteral antibiotics at the time of discharge or else by using study day 14 as an end point, whichever is sooner.
- Receipt of any alternative non-vancomycin anti-MRSA pneumonia antibiotics
  - Defined as linezolid or ceftaroline (daptomycin excluded given inappropriate antibiotic selection for pneumonia).
- Ventilator-free days
  - Defined as the number of days out of the 14 days following enrollment in the trial that the patient is alive and not intubated.
- Total cost of vancomycin use
  - Defined as the estimated cost of vancomycin administration as a composite of cost of drug, cost of associated laboratory testing, and cost of pharmacy services required to provide dosing.
- AKI
  - Defined as an increase in creatinine to ≥ 1.5 times the reference creatinine level or an increase by ≥ 0.3 mg/dL within 14 days of study enrollment. Excludes patients who have ESRD prior to study enrollment.
- In-hospital all-cause mortality
  - Defined as mortality at any point during the hospital stay prior to discharge.
- Predictive value/concordance with final culture-based results

  - Among patients with both a MRSA nasal swab PCR and a respiratory culture at any point from admission to 7 days after study enrollment, percentage of patients with a positive MRSA nasal swab PCR who also had any respiratory culture during the first 7 days after study enrollment that was positive for MRSA (Positive Predictive Value) and percentage of patients with a negative MRSA nasal swab PCR who also had any respiratory culture during the first 7 days after study enrollment that negative for MRSA (Negative Predictive Value).

9.0 Risks and Benefits

9.1 Risks

Given that the only test that will be performed solely for this study is a nasal swab, there is very little direct risk of harm. It is unlikely, however not impossible, that the use of the MRSA nasal swab PCR results to guide vancomycin use will result in inappropriate discontinuation of vancomycin in a patient with MRSA pneumonia. Any risks associated with the receipt of vancomycin would be in line with the standard treatment practices, and not be a result of this study.

Given the collection of protected health information (PHI) for this study, there is the potential for a breach of confidentiality. To mitigate that risk, a minimal amount of PHI will be collected and will be stored in a secure online REDCap database. Only Key Study Personnel will have access to this database containing PHI. Once the data collection period is complete, a research database will be created for data analysis and will be stored on a password protected VUMC server. After data analysis is complete, PHI will no longer be accessed.

9.2 Benefits

This study has the potential to provide a direct benefit to patients by potentially leading to earlier discontinuation of unnecessary antibiotics and thereby decreasing the risk of adverse events. Furthermore, this study will provide a societal benefit by determining the potential merits of implementation of a PCR-based nasal swab screening test.

10.0 Reporting of Adverse Events or Unanticipated Problems

As this study will only utilize diagnostic testing to provide guidance and does not involve any specific treatment or procedural intervention, we do not anticipate any adverse events (AE) related to this study. However, for the purpose of this study, an AE would entail any instance where vancomycin was discontinued based on the results of false negative PCR testing (defined for this study as a negative MRSA nasal swab in instances where a patient is subsequently determined to have a MRSA CAP as evidenced by growth of MRSA from a bacterial blood or respiratory culture collected within 48 hours of the MRSA nasal swab). In particular, any AE which results in or is associated with death, is life-threatening, prolongs hospitalization, or triggers a medical event or intervention to prevent one of these from occurring, will be considered a serious AE (SAE). If any AEs are identified, study personnel will monitor the safety of subjects and follow the AE until the event resolves or is explained.

In order to ensure proper and timely reporting of all adverse events, there will be a clear communication plan for all study personnel to follow. Related or unexpected AEs will be recorded in the AE CRF in the electronic database and reported to the PI within 5 days of occurrence. The PI will provide a report of all related or unexpected AEs that occur annually to the IRB as part of the annual review process. All related or unexpected SAEs will be reported to the PI within 72 hours of occurrence. The PI will, in turn, report all unexpected deaths, serious and treatment related adverse events, and serious and unexpected suspected adverse reactions to the IRB within 7 days after receipt of the report. A written report will be sent to the IRB within 15 calendar days.

11.0 Study Withdrawal/Discontinuation

Patients can be withdrawn from the study if it is retrospectively determined that they did not meet the inclusion criteria or met one of the exclusion criteria. Patients can also be withdrawn if it is determined that there was a significant protocol violation. All withdrawals and the indication for withdrawal will be recorded in the study records. Additionally, follow-up will continue if a patient is withdrawn after the nasal swab test results are reported in order to identify if there are any AEs from false negative PCR testing.

12.0 Statistical Considerations

12.1 Statistical analysis

Regarding statistical analysis procedures, the statistical analysis plan supersedes the methods described in this protocol. Primary and secondary outcomes will be analyzed using an intention-to-treat analysis. The primary outcome for this study will be number of hours alive and free of the use of vancomycin in the 7 days following enrollment. This outcome will be compared between study arms using a longitudinal proportional odds model, with adjustment for baseline covariates. The outcome is derived from a longitudinal model of hourly patient status in order to handle death as an absorbing (terminal) state. Secondary and exploratory outcomes will also be modeled using the flexible family of generalized linear models with similar covariate adjustment. Fidelity to obtaining nasal swabs, running and reporting the rapid PCR results, and sending the pager alert to the clinical team will be evaluated. The potential for differential treatment effects based on fidelity to the expected process of care may be explored.

12.2 Sample size calculations

A 24-hour reduction is considered clinically meaningful.

To overcome challenges with mortality, which we estimate to be 22% within 8 days, we will calculate 'Vancomycin-free hours alive' as the number of hours alive and free of the use of vancomycin within the first seven days of study enrollment. This results in a scale that is best analyzed with a proportional odds model and treatment effect can be summarized as a common odds ratio.

In those who do not die, it is estimated that the relative frequencies for all possible outcomes will be equal to 0.023, 0.008, 0.019, 0.027, 0.095, 0.125, 0.224, 0.262 (for 0 to 7 Vancomycin-free days). Given this distribution, and an assumption of 22% mortality, an overall 24-hour increase in mean 'Vancomycin-free hours alive' would be associated with a common odds ratio for a proportional odds logistic model of about 2.2. To detect this difference with 90% power would require about 106 subjects per study group.

Near the midpoint of the trial, we determined a subset of patients (approximately 12% while being blinded to results) who had a MRSA PCR swab ordered prior to enrollment, and another subset of patients (approximately 3%) who had been co-enrolled in a similar VUMC study evaluating vancomycin use. As these two populations could influence vancomycin use in both the control and intervention arms of the current study, we increased the sample size to account for this contamination, plus one additional patient to account for an instance of mis-

enrollment where the patient did not have an order placed for vancomycin. Based on current incidence rates, the estimated sample size to account for these groups would be 246 patients, with 123 patients per arm. However, as the final percent contamination of these two subgroups within the study's patient population may fluctuate over the remainder of the study period, additional patients will be enrolled as appropriate until the lowest enrolled arm reaches 106 patients in the adjusted cohort, up to a maximum of 300 patients.

12.3 Baseline Characteristics

Baseline characteristics regarding subjects in the study including demographics (age, sex), immunocompromised/immunosuppressed status, pre-existing chronic kidney disease (CKD) or end stage renal disease (ESRD), severity of illness (SOFA score, requirement for supplemental oxygen and/or intubation), and diagnostic microbiological workup (number of blood cultures, respiratory cultures, and other respiratory pathogen specific tests ordered per person) will be reported to determine the effectiveness of randomization.

12.4 Primary Outcome

The primary outcome for this study will be Vancomycin-free hours alive, defined as the number of hours out of the seven days following enrollment in the trial that the patient is alive and not receiving vancomycin. Vancomycin-free hours alive will be analyzed using an intention-to-treat analysis and will use a first-order Markov ordinal longitudinal proportional odds state transition model with three levels of patient status assessed hourly for 7 days (168 hours). The Markov ordinal model will be used to estimate hourly probabilities of being in any of the three states, and these are summed to estimate mean hours within a chosen state such as alive and vancomycin-free. Vancomycin-free hours alive is not computed on a per-patient basis.

12.5 Secondary Outcomes

Multiple secondary outcomes will be evaluated including:

- Time Alive off Vancomycin- The number of hours out of the 168 hours (7 days) following enrollment in the trial that the patient is alive and not receiving vancomycin. Time receiving vancomycin will be defined as the difference from time of administration of the first dose of vancomycin to time of administration of the last dose of vancomycin. If the patient was discharged prior to study day 7 and received vancomycin within 24 hours of discharge then the total duration of vancomycin will be calculated based on the anticipated last day of vancomycin at the time of discharge or else by using study day 7 as an end point, whichever is sooner.

- 30-day all-cause mortality- Defined as mortality within 30 days with date of study enrollment as day 0.

12.6 Exploratory Outcomes

Multiple exploratory outcomes will be evaluated including:

- Length of stay (LOS)- Defined as the difference in days from date of presentation to VUMC to the date of discharge, inclusive of first and last dates.
- Total ICU LOS- Defined as the difference in hours from time of admission to MICU to transfer out of MICU (summed if multiple separate transfers to MICU during the same hospitalization).
- Total duration of antibiotic exposure- Difference in days from time of first dose of any oral or IV antibiotic to the last dose of antibiotics, inclusive of first and last dates. If the patient was discharged prior to study day 14 and received antibiotics within 24 hours of discharge then the total duration of antibiotics will be calculated based on the anticipated last day of antibiotics at the time of discharge or else by using study day 14 as an end point, whichever is sooner.
- Total duration of parenteral antibiotic exposure- Difference in days from time of first dose of any parenteral antibiotic to the last dose of parenteral antibiotics, inclusive of first and last dates. If the patient was discharged prior to study day 14 and had received parenteral antibiotics within 24 hours of discharge then the total duration of parenteral antibiotics will be calculated based on the anticipated last day of parenteral antibiotics at the time of discharge or else by using study day 14 as an end point, whichever is sooner.
- Ventilator-free days- Defined as the number of days out of the 14 days following enrollment in the trial that the patient is alive and not intubated.
- Total cost of vancomycin use- Defined as the estimated cost of vancomycin administration as a composite of cost of drug, cost of associated laboratory testing, and cost of pharmacy services required to provide dosing.
- AKI- Defined as an increase in creatinine to ≥ 1.5 times the reference creatinine level or an increase by ≥ 0.3 mg/dL within 14 days of study enrollment. Excludes patients who have ESRD prior to study enrollment.
- Receipt of any alternative non-vancomycin anti-MRSA pneumonia antibiotics- Defined as receipt of linezolid or ceftaroline during

hospitalization (daptomycin excluded given inappropriate antibiotic selection for pneumonia).

- In-hospital all-cause mortality- Defined as mortality at any point during the hospital stay prior to discharge.
- Predictive value/concordance with final culture-based results- Among patients with both a MRSA nasal swab PCR and a respiratory culture at any point from admission to 7 days after study enrollment, percentage of patients with a positive MRSA nasal swab PCR who also had any respiratory culture during the first 7 days after study enrollment that was positive for MRSA (Positive Predictive Value) and percentage of patients with a negative MRSA nasal swab PCR who also had any respiratory culture during the first 7 days after study enrollment that was negative for MRSA (Negative Predictive Value).

Statistically significant differences in secondary and exploratory outcomes will be calculated using either the Wilcoxon-Mann-Whitney non-parametric test for differences, the Chi-squared test, or Cox proportional hazards model with consideration given to the competing risk of mortality, whenever appropriate.

13.0 Privacy/Confidentiality Issues

A minimal amount of PHI will be collected. PHI will be stored in a secure online REDCap database. Only Key Study Personnel will have access to this database containing PHI. Once the data collection period is complete, a de-identified research database will be created for data analysis and will be stored on a password protected VUMC server indefinitely. After data analysis is complete, PHI will no longer be accessed.

14.0 Follow-up and Record Retention

Patients will be followed for 30 days or until hospital discharge, whichever is longer. PHI will be stored in a secure online REDCap database. Only Key Study Personnel will have access to this database containing PHI. Once the data collection period is complete, a de-identified research database will be created for data analysis and will be stored on a password protected VUMC server indefinitely. After data analysis is complete, PHI will no longer be accessed.

15.0 References

1. Jernigan JA, Hatfield KM, Wolford H, Nelson RE, Olubajo B, Reddy SC, McCarthy N, Paul P, McDonald LC, Kallen A, Fiore A, Craig M, Baggs J. 2020. Multidrug-Resistant Bacterial Infections in U.S. Hospitalized Patients, 2012-2017. N Engl J Med 382:1309-1319.

2. Klein EY, Jiang W, Mojica N, Tseng KK, McNeill R, Cosgrove SE, Perl TM. 2019. National Costs Associated With Methicillin-Susceptible and Methicillin-Resistant Staphylococcus aureus Hospitalizations in the United States, 2010-2014. Clin Infect Dis 68:22-28.

3. Self WH, Wunderink RG, Williams DJ, Zhu Y, Anderson EJ, Balk RA, Fakhran SS, Chappell JD, Casimir G, Courtney DM, Trabue C, Waterer GW, Bramley A, Magill S, Jain S, Edwards KM, Grijalva CG. 2016. Staphylococcus aureus Community-acquired Pneumonia: Prevalence, Clinical Characteristics, and Outcomes. Clin Infect Dis 63:300-9.

4. Jones BE, Ying J, Stevens V, Haroldsen C, He T, Nevers M, Christensen MA, Nelson RE, Stoddard GJ, Sauer BC, Yarbrough PM, Jones MM, Goetz MB, Greene T, Samore MH. 2020. Empirical Anti-MRSA vs Standard Antibiotic Therapy and Risk of 30-Day Mortality in Patients Hospitalized for Pneumonia. JAMA Intern Med 180:552-560.

5. Tongsai S, Koomanachai P. 2016. The safety and efficacy of high versus low vancomycin trough levels in the treatment of patients with infections caused by methicillin-resistant Staphylococcus aureus: a meta-analysis. BMC Res Notes 9:455.

6. van Hal SJ, Paterson DL, Lodise TP. 2013. Systematic review and meta-analysis of vancomycin-induced nephrotoxicity associated with dosing schedules that maintain troughs between 15 and 20 milligrams per liter. Antimicrob Agents Chemother 57:734-44.

7. Fortuna G, Salas-Alanis JC, Guidetti E, Marinkovich MP. 2012. A critical reappraisal of the current data on drug-induced linear immunoglobulin A bullous dermatosis: a real and separate nosological entity? J Am Acad Dermatol 66:988-94.

8. Madigan LM, Fox LP. 2019. Vancomycin-associated drug-induced hypersensitivity syndrome. J Am Acad Dermatol 81:123-128.

9. Huang V, Clayton NA, Welker KH. 2020. Glycopeptide Hypersensitivity and Adverse Reactions. Pharmacy (Basel) 8.

10. Wilhelm MP. 1991. Vancomycin. Mayo Clinic Proceedings 66:1165-1170.

11. Rybak MJ, Le J, Lodise TP, Levine DP, Bradley JS, Liu C, Mueller BA, Pai MP, Wong-Beringer A, Rotschafer JC, Rodvold KA, Maples HD, Lomaestro BM. 2020. Therapeutic monitoring of vancomycin for serious methicillin-resistant Staphylococcus aureus infections: A revised consensus guideline and review by the American Society of Health-System Pharmacists, the Infectious Diseases Society of America, the Pediatric Infectious Diseases Society, and the Society of Infectious Diseases Pharmacists. Am J Health Syst Pharm 77:835-864.

12. Parente DM, Cunha CB, Mylonakis E, Timbrook TT. 2018. The Clinical Utility of Methicillin-Resistant Staphylococcus aureus (MRSA) Nasal Screening to Rule Out MRSA Pneumonia: A Diagnostic Meta-analysis With Antimicrobial Stewardship Implications. Clin Infect Dis 67:1-7.

13. Dadzie P, Dietrich T, Ashurst J. 2019. Impact of a Pharmacist-driven Methicillin-resistant Staphylococcus aureus Polymerase Chain Reaction Nasal Swab Protocol on the De-escalation of Empiric Vancomycin in Patients with Pneumonia in a Rural Healthcare Setting. Cureus 11:e6378.

14. Huffman V, Andrade DC, Ham J, Brown K, Melnitsky L, Lopez Cohen A, Parmar J. 2020. Impact of Nasal Swabs on Empiric Treatment of Respiratory Tract Infections (INSERT-RTI). Pharmacy (Basel) 8.

15. Woolever NL, Schomberg RJ, Cai S, Dierkhising RA, Dababneh AS, Kujak RC. 2020. Pharmacist-Driven MRSA Nasal PCR Screening and the Duration of Empirical Vancomycin Therapy for Suspected MRSA Respiratory Tract Infections. Mayo Clin Proc Innov Qual Outcomes 4:550-556.

16. Dunaway S, Orwig KW, Arbogast ZQ, Myers ZL, Sizemore JA, Giancola SE. 2018. Evaluation of a pharmacy-driven methicillin-resistant Staphylococcus aureus surveillance protocol in pneumonia. Int J Clin Pharm 40:526-532.

17. Smith MN, Erdman MJ, Ferreira JA, Aldridge P, Jankowski CA. 2017. Clinical utility of methicillin-resistant Staphylococcus aureus nasal polymerase chain reaction assay in critically ill patients with nosocomial pneumonia. J Crit Care 38:168-171.

18. Willis C, Allen B, Tucker C, Rottman K, Epps K. 2017. Impact of a pharmacist-driven methicillin-resistant Staphylococcus aureus surveillance protocol. Am J Health Syst Pharm 74:1765-1773.

19. Baby N, Faust AC, Smith T, Sheperd LA, Knoll L, Goodman EL. 2017. Nasal Methicillin-Resistant Staphylococcus aureus (MRSA) PCR Testing Reduces the Duration of MRSA-Targeted Therapy in Patients with Suspected MRSA Pneumonia. Antimicrob Agents Chemother 61.

20. Diep C, Meng L, Pourali S, Hitchcock MM, Alegria W, Swayngim R, Ran R, Banaei N, Deresinski S, Holubar M. 2021. Effect of rapid methicillin-resistant Staphylococcus aureus nasal polymerase chain reaction screening on vancomycin use in the intensive care unit. Am J Health Syst Pharm 78:2236-2244.

21. Raush N, Betthauser KD, Shen K, Krekel T, Kollef MH. 2022. Prospective Nasal Screening for Methicillin-Resistant Staphylococcus aureus in Critically Ill Patients With Suspected Pneumonia. Open Forum Infect Dis 9:ofab578.

22. Metlay JP, Waterer GW, Long AC, Anzueto A, Brozek J, Crothers K, Cooley LA, Dean NC, Fine MJ, Flanders SA, Griffin MR, Metersky ML, Musher DM, Restrepo MI, Whitney CG. 2019. Diagnosis and Treatment of Adults with Community-acquired Pneumonia. An

Official Clinical Practice Guideline of the American Thoracic Society and Infectious Diseases Society of America. Am J Respir Crit Care Med 200:e45-e67.

Swab Testing to Optimize Pneumonia treatment with empiric Vancomycin (STOP-Vanc)

Statistical Analysis Plan

01/22/2025

Frank E Harrell Jr – Lead Biostatistician   Date: 2025-01-27

Frank E Harrell Jr – Lead Biostatistician   Date: 2024-02-08

Frank E Harrell Jr – Lead Biostatistician   Date: 2023-09-25

Introduction

Methicillin-resistant Staphylococcus aureus (MRSA) is a critical antimicrobial resistant threat. Community-acquired pneumonia (CAP) is a major driver of hospital antibiotic use. However, MRSA is an infrequent cause of community-acquired pneumonia (CAP), accounting for less than 1% of cases. Despite this, MRSA is a frequently feared cause of CAP, which leads to the frequent use of vancomycin, an anti-MRSA antibiotic, in empiric CAP treatment. The use of vancomycin is unfortunately associated with a high risk for toxicity and serious adverse events. Furthermore, vancomycin is a costly antibiotic to use in the hospital as it requires careful monitoring due to its narrow therapeutic range and high risk of toxicity. Therefore, reducing inappropriate use of vancomycin in patients with CAP has the potential to improve patient outcomes while at the same time providing overall cost savings. There is growing data to support the use of MRSA nasal swabs as a screening method to guide de-escalation of vancomycin use in CAP. Furthermore, the use of MRSA detection in nasal swabs is now consistent with guideline-based management of CAP.

This document describes the statistical analysis plan for a single center, pragmatic, randomized clinical trial designed to evaluate the impact of MRSA nasal swab PCR detection along with antimicrobial guidance in reducing inappropriate vancomycin usage in CAP patients. This document has been prepared prior to final data collection and unblinding. It is hypothesized that implementation of screening in conjunction with antimicrobial stewardship guidance on interpretation of the results will lead to reduced use of vancomycin. Furthermore, as secondary aims, it is anticipated that a reduction in vancomycin-associated adverse events and an overall cost-savings will be observed, all without increasing mortality or hospital readmissions.

Population and design considerations

Study Population:

Adult (age greater than or equal to 18) patients admitted/transferred to the Vanderbilt University Medical Center (VUMC) Medical Intensive Care Unit (MICU) who are receiving vancomycin for CAP. All eligible patients will be included and there will be no selection based on sex, race, weight, or other clinical factors. Patients will automatically be considered for enrollment either upon transfer to the MICU with an order for "pharmacy to dose vancomycin" already in place, or if an order for vancomycin and/or a consult for "pharmacy to dose vancomycin" is placed (within 24 hours of admission to unit) for a patient located in the MICU, and if they have not already received topical nasal decolonization during the hospitalization. The reasons for ineligibility of patients screened but not enrolled will be recorded.

Inclusion criteria:

- Adult (age greater than or equal to 18) patients admitted/transferred to the VUMC MICU from the VUMC Emergency Department or from a hospital floor within 48 hours of admission.
- Suspicion for pneumonia on admission (defined as an indication for antibiotics of "respiratory infection" and/or an order for a respiratory culture i.e., sputum culture, tracheal aspirate culture, or bronchoalveolar lavage (BAL) culture).
- No topical nasal decolonization during hospitalization prior to collection of MRSA nasal swab PCR.
- Must match both of the following in either order:

- The patient has been admitted to and physically located in the MICU.
- The patient has received a continuing vancomycin order, or a pharmacokinetics consult for a continuing vancomycin order, no later than 24 hours following their physical admission to the MICU.

Exclusion criteria:

- Hospital stay of longer than 48 hours prior to MICU admission.
- Known to be a prisoner.

Study Design:
This is a single center, pragmatic, randomized clinical trial (pRCT) examining whether reporting the results of a negative rapid PCR back to the provider via a pager alert results in decreased vancomycin utilization for critically ill adults with community-acquired pneumonia when compared with usual care.

Randomization:
For patients who meet the study criteria, as outlined above, and are randomized to the intervention arm of the study, a MRSA nasal swab will be collected upon admission to the MICU prior to topical nasal decolonization and a PCR test will be ordered to be run on the collected MRSA nasal swab (no nasal swab will be collected on patients enrolled in the control arm). Patients will be randomized in a 1:1 fashion on an individual patient level.

Sample Size Considerations:
The method used for sample size estimation is based on a traditional univariate proportional odds model. The actual primary analysis is based on a longitudinal proportional odds model that makes use of hourly patient status.

A 24-hour reduction is considered clinically meaningful.

To overcome challenges with mortality, which is estimated to be 22% within 8 days, the primary outcome, 'Vancomycin-free hours alive', will be defined as the number of hours alive and free of the use of vancomycin within the first seven days of study enrollment. This is not a quantity that is calculated on raw data because of deaths but is derived from the longitudinal model described below. The result is summarized as a treatment difference in mean Vancomycin-free hours alive for specific covariate settings (p-values are the same for all covariate settings since treatment is not allowed to interact with covariates). The result will also be summarized by an odds ratio for the hour-to-hour state transitions, from the proportional odds longitudinal model.

In those who do not die, it is estimated that the relative frequencies for all possible outcomes will be equal to 0.023, 0.008, 0.019, 0.027, 0.095, 0.125, 0.224, 0.262 (for 0 to 7 Vancomycin-free days). Given this distribution, and an assumption of 22% mortality, an overall 24-hour increase in mean 'Vancomycin-free hours alive' would be associated with a common odds ratio for a univariate proportional odds logistic model of about 2.2. To detect this difference with 90% power would require about 106 subjects per study group.

Near the midpoint of the trial, we determined a subset of patients (approximately 12% while being blinded to results) who had a MRSA PCR swab ordered prior to enrollment, and another subset of patients (approximately 3%) who had been co-enrolled in a similar VUMC study evaluating vancomycin use. As these two populations could influence vancomycin use in both the control and intervention arms of the current study, we increased the sample size to account for this contamination, plus one additional patient to account for an instance of mis-enrollment where the patient did not have an order placed for vancomycin. Based on current incidence rates, the estimated sample size to account for these groups would be 246 patients, with 123 patients per arm. However, as the final percent contamination of these two subgroups within the study's patient population may fluctuate over the remainder of the study period, additional patients will be enrolled as appropriate until the lowest enrolled arm reaches 106 patients in the adjusted cohort, up to a maximum of 300 patients.

Interventions

a. Control group: Enrolled patients in which no MRSA nasal swab is collected and PCR is not run.
b. Intervention group: Enrolled patients in which the MRSA nasal swab PCR will be run and once the MRSA nasal swab PCR has been completed, the results will be available in the EMR along with antimicrobial stewardship program guidance. For patients in the study group with a negative MRSA nasal swab PCR, a pager alert will be sent to the patient's primary team. The pager alert will inform the provider of the negative test result and will direct them to the result in the EMR where they will receive further clinical guidance recommending the provider consider discontinuing vancomycin. In addition to the direct, instantaneous antimicrobial stewardship guidance to be delivered through the EMR as outlined above, general education about the use of MRSA nasal swab PCR testing to guide de-escalation of empiric vancomycin will be provided by the MICU pharmacist when a patient with a negative MRSA nasal swab PCR is continued on vancomycin.

Endpoints

Primary Endpoint

The primary outcome is ordinal 3-level patient status assessed on 168 consecutive hours, with the ordinal status levels being alive and not on Vancomycin, alive and on Vancomycin, or dead. The statistical model explicitly considers death as a negative outcome that is a terminal event (absorbing state in state transition model terminology). The underlying statistical parameter representing the treatment effect is an odds ratio (OR) for staying or transitioning to states given the previous day's state. An OR greater than 1.0 represents higher odds of Vancomycin or death and higher odds of death conditional on the previous day's state (which can only be one of the first two of the three possible states). The OR is translated to clinical restatements of the analysis model as follows (p-values from these other estimands are identical to the p-value from the underlying OR). The ordinal longitudinal transition model directly provides transition probabilities, and the law of total probability is used to convert these to unconditional probabilities (state occupancy probabilities - SOPs) such as the probably of being alive and Vancomycin-free on the 48th hour. The SOPs are summed over hours to estimate mean time in

state, which is used to provide the estimate of the treatment difference in the mean number of Vancomycin-free hours alive, which will be the first estimate reported.

Secondary Endpoint(s)
There are two prespecified secondary outcomes for this trial.

Time Alive off Vancomycin: The number of hours out of the 168 hours (7 days) following enrollment in the trial that the patient is alive and not receiving vancomycin. Time receiving vancomycin will be defined as the difference from time of administration of the first dose of vancomycin to time of administration of the last dose of vancomycin. If the patient was discharged prior to study day 7 and received vancomycin within 24 hours of discharge then the total duration of vancomycin will be calculated based on the anticipated last day of vancomycin at the time of discharge or else by using study day 7 as an end point, whichever is sooner.

30-day all-cause mortality: Defined as mortality within 30 days with date of study enrollment as day 0.

Exploratory Endpoint(s)
There are multiple prespecified exploratory outcomes for this trial.

Length of stay (LOS): Defined as the difference in days from date of presentation to VUMC to the date of discharge, inclusive of first and last dates.

Total ICU LOS: Defined as the difference in hours from time of admission to MICU to transfer out of MICU (summed if multiple separate transfers to MICU during the same hospitalization).

Total duration of antibiotic exposure: Defined as the difference in days from time of first dose of any oral or IV antibiotic to the last dose of antibiotics, inclusive of first and last dates. If the patient was discharged prior to study day 14 and received antibiotics within 24 hours of discharge then the total duration of antibiotics will be calculated based on the anticipated last day of antibiotics at the time of discharge or else by using study day 14 as an end point, whichever is sooner.
Total duration of parenteral antibiotic exposure: Defined as the difference in days from time of first dose of any parenteral antibiotic to the last dose of parenteral antibiotics, inclusive of first and last dates. If the patient was discharged prior to study day 14 and had received parenteral antibiotics within 24 hours of discharge then the total duration of parenteral antibiotics will be calculated based on the anticipated last day of parenteral antibiotics at the time of discharge or else by using study day 14 as an end point, whichever is sooner.

Ventilator-free days: Defined as the number of days out of the 14 days following enrollment in the trial that the patient is alive and not intubated.

Total cost of vancomycin use: Defined as the estimated cost of vancomycin administration as a composite of cost of drug, cost of associated laboratory testing, and cost of pharmacy services required to provide dosing.

AKI: Defined as an increase in creatinine to ≥ 1.5 times the reference creatinine level or an increase by ≥ 0.3 mg/dL within 14 days of study enrollment. Excludes patients who have end stage renal disease (ESRD) prior to study enrollment.

Receipt of any alternative non-vancomycin anti-MRSA pneumonia antibiotics: Defined as receipt of linezolid or ceftaroline during hospitalization (daptomycin excluded given inappropriate antibiotic selection for pneumonia).

In-hospital all-cause mortality: Defined as mortality at any point during the hospital stay prior to discharge.

Predictive value/concordance with final culture-based results: Among patients with both a MRSA nasal swab PCR and a respiratory culture at any point from admission to 7 days after study enrollment, this outcome is defined as the percentage of patients with a positive MRSA nasal swab PCR who also had any respiratory culture during the first 7 days after study enrollment that was positive for MRSA (Positive Predictive Value) and the percentage of patients with a negative MRSA nasal swab PCR who also had any respiratory culture during the first 7 days after study enrollment that was negative for MRSA (Negative Predictive Value).

Implementation Endpoints
Time from collection of swab to results being available in EHR

Fidelity Endpoint
Fidelity of Obtaining Nasal Swab
Fidelity of Running and Reporting PCR Results
Fidelity of Paging Alert to Team

Analysis dataset
The analysis for the trial will use an intent-to-treat approach to answer the effectiveness question posed. That is, participants will be evaluated by treatment group as assigned regardless of what was delivered. All eligible participants will be included.

Statistical Approach
Our initial analysis will be descriptive in nature, summarizing information that characterizes the cohort and the outcomes. Then, we will proceed with inferential analysis to answer the main study question. Then, we will compare the secondary endpoints between study groups.

Descriptive Analysis
To characterize the study sample, baseline demographic and clinical data will be described overall and by group. Categorical variables will be described using frequencies and proportions, and continuous variables will be described using medians and interquartile ranges. Missingness will be reported for each variable. Graphical summaries using box plots, violin plots, and/or histograms may be used to describe the data graphically. At a minimum, the following variables will be described at time of enrollment:

- Encounter ID (patients could be re-enrolled if they have a second or multiple admissions; each admission should be treated separately)
- Age (years)
- Sex (male, female, unknown)
- Race (African American, Asian/Pacific Islander, Caucasian, Multiple, Native American, Other, Unknown)
- Ethnicity (Hispanic, Non-Hispanic, Unknown)
- Height (m)
- Weight (kg)
- BMI
- Elixhauser comorbidity index
- Pre-existing chronic kidney disease (CKD)
  - Baseline CKD
  - Reference Creatinine - Defined as the most recent creatinine obtained since admission but prior to the administration of the first dose of vancomycin. If no value exists, then the most immediate creatinine following the administration of vancomycin will be used.
- End stage renal disease (ESRD)
- Immunocompromised/immunosuppressed
  - HIV
  - Solid organ transplant recipient
  - Stem cell transplant recipient
- Severity of illness
  - SOFA score
  - Requirement for supplemental oxygen and/or intubation
- Diagnostic microbiological workup
  - Number of blood cultures, respiratory cultures, and other respiratory pathogen specific tests ordered per person

We will describe all of the outcome variables overall and grouped by study arm using the same approach as for the demographic data. Summary statistics and graphical representations may be displayed, and missingness will be reported for each variable.

No statistical comparisons between groups will be done for this descriptive analysis. Please note, showing descriptive statistics stratified by treatment groups that were randomized can be easily misinterpreted, as all apparent imbalances are by definition due to chance. Descriptive statistics of all-comers need to be emphasized in randomized studies.

Main Analysis
Primary and secondary outcomes will be analyzed using an intention-to-treat analysis. The primary analysis will use a first-order Markov ordinal longitudinal proportional odds (PO) state transition model with three levels of patient status assessed hourly for 168 hours as described under *Primary Endpoint* above. This model places death as the worst outcome and does not need to code death numerically. A traditional approach of using Vancomycin-free hours alive with death coded as –1 has interpretation problems (and does not handle missing data) and neither means (because of the arbitrariness of –1) nor medians (because of excessive ties in the data) are

adequate summary statistics. The underlying transition model is a partial PO model, *partial* because the effect of time is allowed to vary with the ordinal state being predicted. Put another way, Vancomycin may be used earlier, and deaths occur later, and it is important for the model not to have restrictions on how the various events unfold over time. The model is adjusted for baseline covariates. The within-patient correlation structure in the 3-level statuses is flexibly handled by a Markov process. “First order” means that each transition is conditional only on the previous day’s state and not statuses that are earlier. The partial PO model will assume PO for all other effects including treatment.

Letting the outcome variable on day t be denoted by Y(t) with levels 0, 1, 2 (alive & Vancomycin-free, alive & on Vancomycin, dead), and letting expit denote the inverse logit transform, the ordinal logistic model for day t may be written as follows, with y=1 or 2.

$$Pr(Y >= y \mid Y(t-1), X) = expit(a(y) + X*beta + t*tau*[y=2] + gamma*[Y(t-1) = 1])$$

Here a(y) is the intercept corresponding to y, X represents baseline covariates, linear time effect, and a binary treatment indicator, beta are the main regression coefficients, and tau is a special effect of time t when y=2 (the partial PO effect). [z] denotes a 0-1 indicator function that is 1 if z is true, 0 otherwise.

A detailed case study using the proposed method, with code and results, may be found at https://hbiostat.org/rmsc/markov.html

The primary analysis will use R version 4.3.1 or later using the R VGAM package to fit the partial PO model and the Hmisc package to compute SOPs. Results will be displayed descriptively as 3-level stacked bar charts by treatment, covariate adjusted.

Likelihood ratio tests will be used for pivotal tests. These have better performance than Wald tests.

We do not expect missingness in our primary outcome. If there are missing covariates, cases will not be excluded; we will use multiple imputation with predictive mean matching for missingness in adjusting covariates.

Secondary and exploratory outcomes will also be modeled using the flexible family of generalized linear models with similar covariate adjustment. Statistically significant differences in secondary and exploratory outcomes will be calculated using either the Wilcoxon-Mann-Whitney non-parametric test for differences, the Chi-squared test, or Cox proportional hazards model with consideration given to the competing risk of mortality, whenever appropriate. Fidelity to obtaining nasal swabs, running the rapid PCR, and delivering results will be evaluated. The potential for differential treatment effects based on fidelity to the expected process of care may be explored.

There may be missingness in secondary or implementation outcomes. The cohort for which the outcome is available will be described, along with the results of the model evaluating treatment effects in this cohort. All model results will be summarized with point estimates and 95%

confidence intervals (CIs), which will be emphasized over p-values when reporting the results for secondary and implementation outcomes. No adjustments for multiplicity will be made.

Additionally, a descriptive analysis of missingness tendencies is warranted. For example, logistic regression to predict the probability of missingness of an outcome measurement with predictors that include baseline variables and other outcomes that are never missing.

Differential treatment effects

To determine whether effects of treatment on the primary endpoint depends on any of the baseline characteristics, we will test the interaction between the baseline characteristics and treatment effect. This is done by adding, one-at-a-time the potential interacting factors to the model detailed above. Our detailed strategy for assessing differential treatment effects may be found at https://hbiostat.org/bbr/ancova.html#modeling-differential-treatment-effect. The prespecified potential interacting factors are as follows, Elixhauser comorbidity index, immunocompromised/immunosuppressed status, baseline CKD, and severity of illness.

Summary

The results of this study will help to determine whether reporting the results of a negative rapid PCR back to the provider, via a pager alert, results in decreased vancomycin utilization for critically ill adults with community-acquired pneumonia when compared with usual care. The analysis approach described is selected based on the trial's pragmatic nature and the intent to understand the effectiveness of MRSA nasal swab PCR screening along with antimicrobial guidance in reducing inappropriate vancomycin usage in patients with community-acquired pneumonia.

Version and Revision Log

9/25/2023	Version 1: developed with and approved by Frank E Harrell, Jr., PhD.
2/8/2024	Version 2: Edits developed and approved by Frank E Harrell, Jr., PhD.<br>Revisions:<br>• Changing the study name.
1/22/2025	Version 3: Edits developed and approved by Frank E Harrell, Jr., PhD.<br>Revisions:<br>• Changing the study sample size<br>• Correcting the logistic regression formula equation, page 8
